# Integrated analysis of independent gene expression microarray datasets improves the predictability of breast cancer outcome

**DOI:** 10.1186/1471-2164-8-331

**Published:** 2007-09-20

**Authors:** Zhe Zhang, Dechang Chen, David A Fenstermacher

**Affiliations:** 1 Department of Biomedical Engineering, University of North Carolina, Chapel Hill NC 27506, USA; 2 Research Informatics, H. Lee Moffitt Cancer Center and Research Institute, Tampa, FL 33612, USA; 3 Department of Preventive Medicine and Biometrics, Uniformed Services University of the Health Sciences, Bethesda, MD 20814, USA

## Abstract

**Background:**

Gene expression profiles based on microarray data have been suggested by many studies as potential molecular prognostic indexes of breast cancer. However, due to the confounding effect of clinical background, independent studies often obtained inconsistent results. The current study investigated the possibility to improve the quality and generality of expression profiles by integrated analysis of multiple datasets. Profiles of recurrence outcome were derived from two independent datasets and validated by a third dataset.

**Results:**

The clinical background of patients significantly influenced the content and performance of expression profiles when the training samples were unbalanced. The integrated profiling of two independent datasets lead to higher classification accuracy (71.11% vs. 70.59%) and larger ROC curve area (0.789 vs. 0.767) of the testing samples. Cell cycle, especially M phase mitosis, was significantly overrepresented by the 60-gene profile obtained from integrated analysis (p < 0.0001). This profiles significantly differentiated poor and good prognosis in a third patient cohort (p = 0.003). Simulation procedures demonstrated that the change of profile specificity had more instant influence on the performance of expression profiles than the change of profile sensitivity.

**Conclusion:**

The current study confirmed that the gene expression profile generated by integrated analysis of multiple datasets achieved better prediction of breast cancer recurrence. However, the content and performance of profiles was confounded by clinical background of training patients. In future studies, prognostic profile applicable to the general population should be derived from more diversified and balanced patient cohorts in larger scale.

## Background

Breast cancer involves a series of genomic disorders, making it a suitable subject of microarray experiments [1]. Mapping microarray-based gene expression profiles to clinical phenotypes has been proposed as a solution to improve cancer diagnosis and prognosis [2]. A number of such profiles, which are able to distinguish cell lines [3], normal and tumor tissues [4], adjacent tumors [5], and tumor subtypes [6,7], have been presented. Expression profiles of cancer endpoints are more valuable in clinical practice. From a microarray dataset of 78 breast cancer patients, van 't Veer *et al *identified a 70-gene profile that correctly classified 5-year recurrence of 65 (83%) patients [8]. This profile was further proved to be superior to currently used indexes [8,9]. Similar profiles were identified by other studies [10-12]. However, these profiles shared little overlap with each other. It was further noticed that highly distinct profiles had similar performance and significant agreement on recurrence prediction [13,14]. These observations indicate that the expression profiling of cancer prognosis is more complicated than simply identifying a list of differentially expressed genes from a single dataset.

Despite of the prospective benefits, key issues related to expression profiling of cancer prognosis still remain in question. First, it should be presumed that the classification of patient prognostic groups properly reflects the inherent difference between their gene expression patterns. Studies usually dichotomize breast cancer patients according to clinically used 5-year prognosis [8,10]. However, this convention is established by usage rather than based upon intrinsic biological difference between tumor cells, and may reduce the statistical power of subsequent analyses. Retsky *et al. *discovered that the recurrence of breast cancer has a two-peak distribution independent of tumor size, number of positive nodes, and menopause status [15]. Computer simulation of tumor progression suggested that two different models of secondary tumor growth were responsible for this distribution [15,16]. The 18-month peak was the consequence of accelerated secondary growth stimulated by mastectomy while patients in the 60-month peak went through steady stochastic transitions of tumor progression phases.

Another issue is the influence of clinical confounders, such as ER and lymph node status. Gruvberger *et al *noticed that 165 of the 231 genes top-ranked in van 't Veer paper were also significantly correlated to ER status of patients [17,18]. It was then suggested that expression profiling should be carried out for ER-positive and -negative patients separately. Expression profile derived from one patient cohort might not be applicable to other cohorts having dissimilar clinical background. Removing or reducing the confounding effect will improve the robustness of expression profiles. Nevertheless, the suggestion of Gruvberger *et al *may not be a practical solution because there are many known and unknown confounders intervening in the correlation between gene expression level and breast cancer recurrence.

Furthermore, comparing to the large number of genes (variables) measured by microarray experiments, sample sizes are usually too small to give enough statistical power. Consequently, gene expression profiles unavoidably include false positives due to 'multiple hypothesis testing' [19] while many differentially expressed genes will not be identified due to lack of statistical power. A question worthy of more discussion is how sensitivity and specificity should be optimally balanced in expression profiles.

Integrated analysis of multiple independent microarray datasets has drawn noteworthy interests recently [20-22]. Not only will this strategy increase the overall statistical power of expression profiling, but also it can reduce the influence of confounders by including diversified samples. Genes directly and consistently, but not obviously, correlated to disease outcome will be preferred by integrated analysis. A basic assumption of integrated analysis is that independently generated datasets may share common information despite of systematic variations between experiments. Ghosh *et al *investigated the consistence of four independent microarray datasets from prostate cancer [21]. Meta-analysis of those datasets concluded that their gene expression profiles are significantly similar to each other. Rhodes *et al *compared the expression profiles of normal and tumor cells in a larger scale using 21 datasets from 12 tissue types [22]. 67 genes consistently correlated to the normal-tumor phenotypes across datasets were proposed as a generic expression profile of neoplastic transformation.

The aim of this study is to improve the expression profiling of breast cancer recurrence by integrating independent datasets. Breast cancer patients were classified according to Retsky recurrence model. Expression profiles derived from two individual datasets and their integration were objectively compared by random re-sampling and cross-validation. It was demonstrated that the expression profiles had higher specificity after datasets were integrated. Furthermore, the resultant expression profiles were validated by a third dataset.

## Results

### SEP (Score for Expression Profile) as a prognosis index of breast cancer

**Figure 1:**
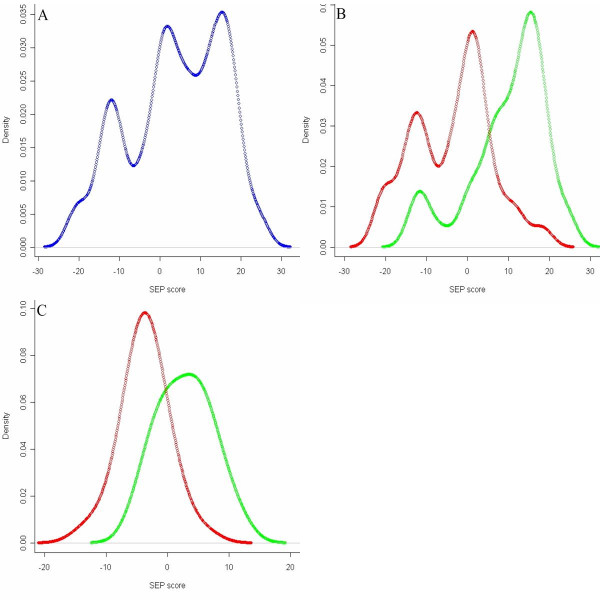
**Density distribution of SEP scores**. SEP of breast cancer patients was calculated with microarray data published by van' t Veer *et al*. (A) The distribution of all 78 scores was plotted altogether. SEP was calculated with 231 genes correlated to 5-year recurrence outcome with |r| > 0.3 according to the original study. (B) The score distributions of 44 good prognosis patients and 34 prognosis patients were separately plotted. Good prognosis patients were expected to have higher SEP scores. (C) The score distributions of good and poor prognosis patients were plotted again after confounding effect of ER status was removed by partial correlation analysis. SEP was calculated with 127 genes correlated to 5-year outcome with |r'| > 0.3.

According to the original study using Rosetta dataset, the expression level of 231 genes was significantly correlated (|r| > 0.3, p < 0.01) to 5-year recurrence of 78 breast cancer patients [8]. SEP scores were calculated using those patients and genes while the expected expression level (E_i_) of each gene in formula (1) was set to zero and the weight (w_i_) was set to the Pearson correlation coefficient (r) between gene expression and 5-year recurrence outcome [see Additional file 1]. Fig. 1A plots the density distribution of resultant 78 scores, which has a surprising three-peak model. The score distributions of two prognosis groups are separately plotted in Fig. 1B, which indicates that the right and middle peaks in Fig. 1A are mainly composed of poor and good prognosis patients respectively while the left peak was a mixture of both. It was also noticed that patients having the lowest SEP scores (left peak) were mostly ER-negative (11 of 13) while the other patients were mostly ER-positive (54 of 65).

Fisher's exact test was used to evaluate the dependence of SEP on major clinical indexes after patients were equally separated into high score and low score groups with the threshold equal to median score. The results showed that the values of SEP were significantly dependent on ER status (positive vs. negative), PR status (positive vs. negative), tumor size (T1 vs. T2) and histological grade (1 vs. 2 vs. 3) with p < 0.001, but not on angioinvasion (positive vs. negative, p = 0.21) or age of patients (<= 40 vs. >40, p = 0.61).

Partial correlation analysis was then applied to control out the confounding effect of ER status. Correlation between recurrence outcome and residuals obtained from Formula (2) was calculated and the 127 genes having |r'| > 0.3 were used to recalculate SEP scores. The score distributions of two prognosis groups are separately plotted in Fig. 1C. Results of Fisher's exact test showed that modified SEP was not dependent on ER status (p = 0.21), but still significantly dependent on histological grade (p < 0.001) and tumor size (p = 0.006), and marginally on PR status (p = 0.04).

### Analysis of two independent datasets

The current study incorporated permutated re-sampling, training/testing validation, and stepwise procedure to objectively compare performance of prognostic expression profiles. The workflow was first applied to Rosetta and Stanford datasets separately. Patients in each prognosis group of each dataset were randomly re-sampled into training (about two-thirds of total patients) and testing (the rest patients) subgroups. The expression profiles were generated from the training patients and tested by the testing patients. To avoid sampling bias, patients were repeatedly re-sampled to obtain different combinations of training/testing subgroups upon which the following analytical steps were repeated. After each re-sampling, the differential expression of each gene between two prognosis groups was tested by non-parametric Wilcoxon Rank Sum Test (RST) [23] using the training data and the resultant Z statistics was used to rank all 5,569 genes. The gene whose Z value had the largest magnitude was ranked the highest. Top-ranked N genes constituted an expression profile. Increasing the value of N would supposedly improve profile sensitivity, but reduce specificity at the same time. A stepwise procedure was carried out to find the optimal balance between specificity and sensitivity of profiles, during which top-ranked genes were added one by one until N = 100. Testing patients were re-scored at each step using Formula (1), while the weight of each gene equal to its Z statistic and the expected value equal to the average expression of that gene in training patients. Testing patients were classified into two groups using resultant SEP scores (positive vs. negative). The SEP-based classification was matched to actual recurrence outcome to get its accuracy. To take advantage of SEP as a continuous variable, scores were also used to build ROC curve and the area under the curve (AUC) indicated how much the prognosis groups were differentiated by SEP.

Previous steps were repeated for 10,000 re-samplings. The upper half of Table 1 shows the median classification accuracy and ROC curve area achieved by individual datasets when N was 100. At each re-sampling, the overall accuracy and AUC of two datasets were also calculated as size (of testing subgroups)-weighted averages. The 90% CI of size-weighted average accuracy and AUC was (61%, 80%) and (0.69, 0.88) respectively. Only eight re-samplings got less than 50% accuracy, giving a permutation p value equal to 0.0008. Rosetta dataset obtained generally better results than Stanford dataset, probably because of its relatively larger sample size and less diverse clinical background of patients.

**Table 1:** Predictability of expression profiles

**Training dataset**	**Tested on Rosetta**		**Tested on Stanford**		**Size-weighted average**	
	Accuracy	AUC	Accuracy	AUC	Accuracy	AUC

Individual	71.43%	0.775	70.00%	0.764	70.59%	0.767
Combined	71.43%	0.786	71.43%	0.799	71.11%	0.789

At each permutation re-sampling, SEP of testing patients was calculated with top-ranked genes obtained from training patients; the mean of accuracy and AUC was weighted by size of testing subgroups; values presented were the medians summarized from10,000 re-samplings.

**Figure 2:**
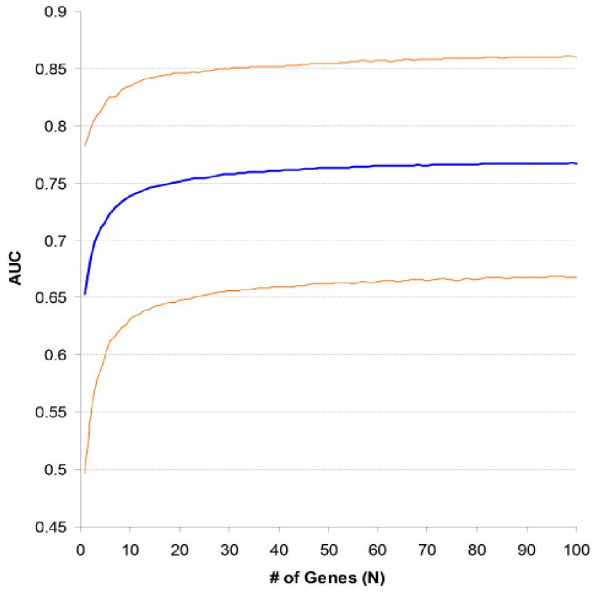
**Changing of ROC curve area with size of expression profiles**. Permutation median (blue line) and 90% CI (orange lines) of ROC curve area summarized from two independent datasets and 10,000 re-samplings. ROC curves were built with SEP scores of testing patients at each re-sampling while SEP was calculated with expression profiles identified from the training patients. The size of expression profiles was increased from 1 to 100 one by one. Average curve area of two testing subgroups was weighted by their size.

Fig. 2 plots the median and 90% CI of size-weighted average AUC when N was 1 till 100. The median AUC went up dramatically at the beginning of the curve and approached a plateau when N was about 60, suggesting that the differential ability of expression profiles was about to be saturated. Since increasing N would also increase the ratio of false positives in profiles, it was empirically decided that the sensitivity and specificity of expression profiles were optimally balanced at N = 60. Except when N was very small, the scale of 90% CI did not change noticeably with N.

A final gene ranking was obtained from each dataset based on how many times each gene was ranked within top 100 through all re-samplings. The 60 genes having the largest counts constituted an ultimate expression profile of a dataset. When these genes were used to calculate SEP scores, the weight was their RST Z statistics calculated from all patients in that dataset and the expected expression was conservatively set to 0 by default. The 60-gene profiles of both datasets were precise classifiers when they were self-validated. Rosetta profile had an accuracy of 79.3% and AUC of 0.89, and Stanford profile achieved 82.3% and 0.93 respectively. Although the two profiles only shared two common genes, BUB1 and LRP8, they were both cross-validated by the other dataset as satisfying predictors of 3-year recurrence (Table 2).

**Table 2:** Cross-validation of two independent datasets

**Profile**	**Validated by**	**Accuracy**	**AUC**	**Odds ratio**
Rosetta	Stanford	74.2%	0.808	9.06
Stanford	Rosetta	70.7%	0.786	6.68
Overall		72.2%	0.795	6.71

SEP scores of validating patients were calculated with the 60-gene profile of the validated dataset. Patients were evenly classified into two groups using median score as threshold. Accuracy: percentage of correctly classified patients; AUC: area of SEP-based ROC curve; Odds ratio: the association between SEP and disease outcome based on Fisher's exact test.

The validating SEP was compared to currently used prognostic indexes of breast cancer with logistic regression models. Table 3 compares the fitness of each model. When independent variables were used individually, models built using SEP had the best fitness, followed by models of histological grade. Multivariate models were built with all available indexes as independent variables. Likelihood ratio tests showed that when SEP was removed from the multivariate model of Rosetta dataset, the model performance was significantly reduced (p = 0.006); but did not get similar result from Stanford data (p = 0.15), probably due to smaller number of samples.

**Table 3:** Comparison of prognostic indexes with logistic regression models

	**Rosetta**			**Stanford**		
	**-2LL**	**L. R.**	**p value**	**-2LL**	**L. R.**	**p value**

Intercept	108.7			79.9		
SEP	93.1	15.6	0.0001	65.9	14.0	0.0002
ER status	98.0	10.8	0.001	74.3	5.5	0.02
PR status	103.5	5.3	0.02	NA	NA	NA
Tumor size	101.2	7.6	0.006	76.6	3.3	0.35
Lymph node status	NA	NA	NA	78.2	1.7	0.44
Histological grade	92.9	15.9	0.0004	70.4	9.5	0.009
Age at diagnosis	98.9	9.8	0.002	78.6	1.24	0.27
Angioinvasion	104.4	4.4	0.04	NA	NA	NA
Multivariate model (include SEP)	67.7	41.0	0.000002	54.8	25.1	0.005
Multivariate model (not include SEP)	75.2	33.6	0.00002	56.9	23.0	0.006

Model fitness was represented by -2LL (-2 log likelihood), L.R. (likelihood ratio), and p value. Multivariate models were built with all presented indexes with or without SEP. NA: value of index was uniform or not given. Two Stanford samples, svcc76 and svn007, were not included because of incomplete clinical information.

**Figure 3:**
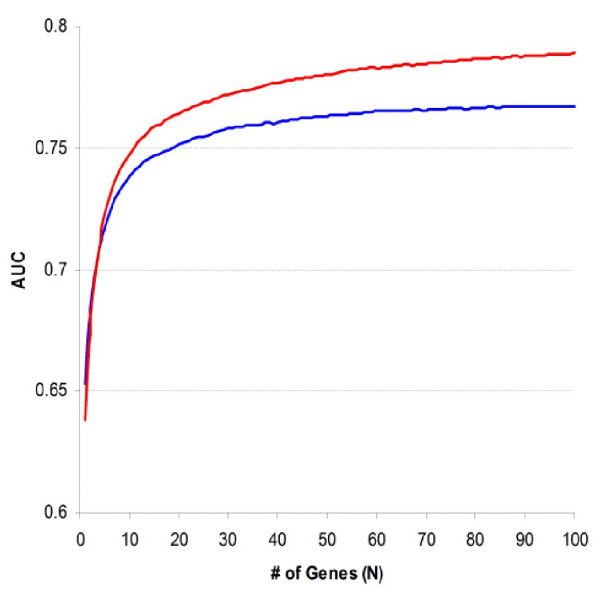
**Comparison of profiles from individual and combined datasets**. ROC curves were built with testing SEP scores obtained from two individual datasets and their combination. The size of expression profiles was increased from 1 to 100 one by one. Median areas were summarized from 10,000 re-samplings. Size-weighted average areas achieved by the combined dataset (red line) were generally larger than the corresponding areas achieved by individual datasets (blue line).

The next analysis of this study was to integrate Rosetta and Stanford datasets. After each re-sampling as described above, training patients from both datasets were pooled together and RST was performed on each gene using the combined data. Genes were ranked by their Z statistics and top-ranked ones were used to constitute expression profiles, which would be validated separately by testing patients of individual datasets. Table 1 summarizes the median of classification accuracy and AUC with N = 100 and the sized-weighted averages of these statistics were generally higher than those obtained from the individual datasets. The median accuracy was slightly raised by 0.52% and the median AUC was more notably raised by 0.022. Five of 10,000 re-samplings got less than 50% classification accuracy, giving a permutation p value of 0.0005. Fig. 3 plots the median of size-weighted average AUC against the value of N. The combined dataset always achieved larger median AUC than the individual ones when N > 3. At N = 100, the profiles of the combined dataset outperformed those of individual datasets in about 74% re-samplings.

**Figure 4:**
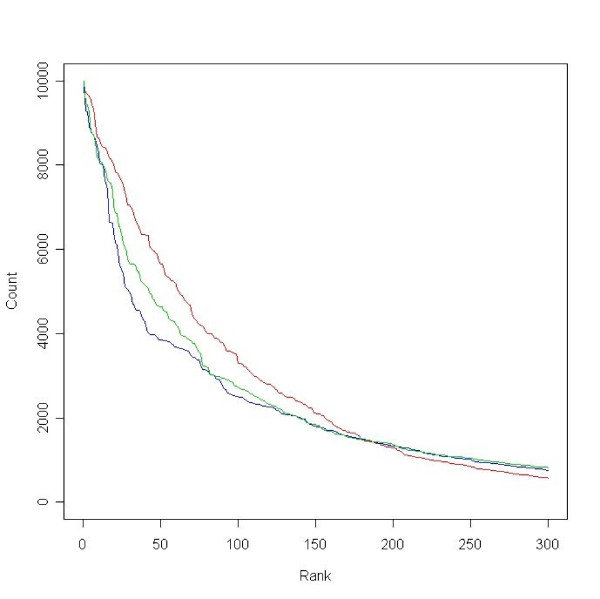
**Consistence of gene ranking across re-samplings**. The counts by which each gene was ranked in top-100 across 10,000 re-samplings were summarized from both individual datasets and the combined dataset. Genes were ranked based on their counts and the counts of top-ranked 300 genes were presented (blue line: Stanford dataset, green line: Rosetta dataset, red line: the combined dataset).

The count of each gene ranked within top-100 by the combined dataset was also used to rank genes. Fig. 4 demonstrates the counts of 300 top-ranked genes by two individual datasets and the combined dataset. It shows that the repeatability of ranking genes was generally low. For example, the 100^th ^gene had less than one-third probability to be ranked top-100 by all three datasets. The combined dataset ranked genes relatively more consistently. The 60^th ^gene was ranked within top-100 by more than half of the permutations.

Some genes in the 60-gene profiles of three different datasets are listed in Table 4 while the complete gene lists are provided as supplementary data [see Additional file 2]. Two genes (BUB1 and LRP8) were presented in all profiles while fifteen others (MPL, BECN1, etc.) were only in the combined dataset profile. These genes were ranked higher by the combined dataset because of their low inter-dataset variance. The combined dataset profile included two known molecular markers of breast cancer: BCL2 and ESR1.

**Table 4:** Focus genes in expression profiles of breast cancer outcome

**Unigene**	**Symbol**	**Full name**	**Combined (count/rank)**	**Rosetta (count/rank)**	**Stanford (count/rank)**
Hs.469649	BUB1	BUB1 budding uninhibited by benzimidazoles 1 homolog	3/9,681	24/6,547	11/8,047
Hs.576154	LRP8	low density lipoprotein receptor-related protein 8	13/8,428	60/4,211	50/3,858
Hs.496068	PCTK1	PCTAIRE protein kinase 1	1/9,862	5/8,768	120/2,261
Hs.523468	SCUBE2	signal peptide, CUB domain, EGF-like 2	2/9,732	1/9,991	3,615/1
Hs.523836	GSTP1	glutathione S-transferase pi	228/989	>3,257/0	1/9,866
Hs.208124	ESR1	estrogen receptor 1	4/9,647	248/1,048	2/9,297
Hs.58974	CCNA2	cyclin A2	9/8,673	698/149	4/8,925
Hs.524134	GATA3	GATA binding protein 3	10/8,651	99/2,755	25/5,530
Hs.82906	MPL	Myeloproliferative leukaemia virus oncogene	12/8,466	120/2,329	67/3,606
Hs.267659	VAV3	vav 3 oncogene	14/8,415	284/881	20/6,421
Hs.12272	BECN1	beclin 1 (coiled-coil, myosin like BCL2 interacting protein)	18/8,158	74/3,603	96/2,560
Hs.153752	CDC25B	cell division cycle 25B	24/7,690	37/5,249	127/2,140
Hs.9589	UBQLN1	ubiquilin 1	25/7,634	7/8,723	824/145
Hs.150749	BCL2	B-cell CLL/lymphoma 2	30/7,047	750/126	13/8,003
Hs.69771	CFB	Complement factor B	60/5,168	320/738	35/4,561
Hs.182385	HPN	hepsin (transmembrane protease, serine 1)	1,103/16	>3,257/0	60/3,685

Count: how many times a gene was ranked within top-100 by 10,000 re-samplings; Rank: rank of the gene based on its count.

Among the 60 genes of each profile, the least significant p value of RST was 0.0002 (Rosetta), 0.0014 (Stanford), or 0.00002 (Combined), respectively corresponding to false discovery rates 0.017, 0.097, or 0.006. The improvement achieved by the combined dataset indicated that more statistical power was gained by data integration.

The DAVID functional annotation tool [24] was applied to the combined dataset profile while all 5,569 Unigene clusters were used as the genomic background. Some of the enriched gene sets are listed in Table 5. Cell Cycle, especially M Phase Mitosis, was the most significantly overrepresented gene set. Without redundancy, 35 genes in the profile were included by the gene sets in Table 5. A complete list of these genes and other enriched gene sets are available as supplementary materials [see Additional file 3].

**Table 5:** Gene sets enriched in the combined dataset profile

**Category**	**Gene set**	**Count**	**p value**
GO:Biological Process	Cell cycle/M phase of mitotic cell cycle	14/8	0.00008/0.00001
BIND	Myosin, heavy polypeptide 10, non-muscle	5	0.01
BIND	E2F transcription factor 5, P130 binding	2	0.03
GO:Biological Process	Negative regulation of apoptosis	4	0.05
GO:Biological Process	Signal transduction	18	0.06
GO:Cellular Component	Microtubule cytoskeleton	4	0.07
GO:Molecular Function	Enzyme regulator activity	7	0.09

Category: database resource of the gene set; Count: number of genes overlapped by the 60-gene profile and the gene set; p value: result of Fisher's exact test.

### Validation of profiles with a third dataset

**Figure 5:**
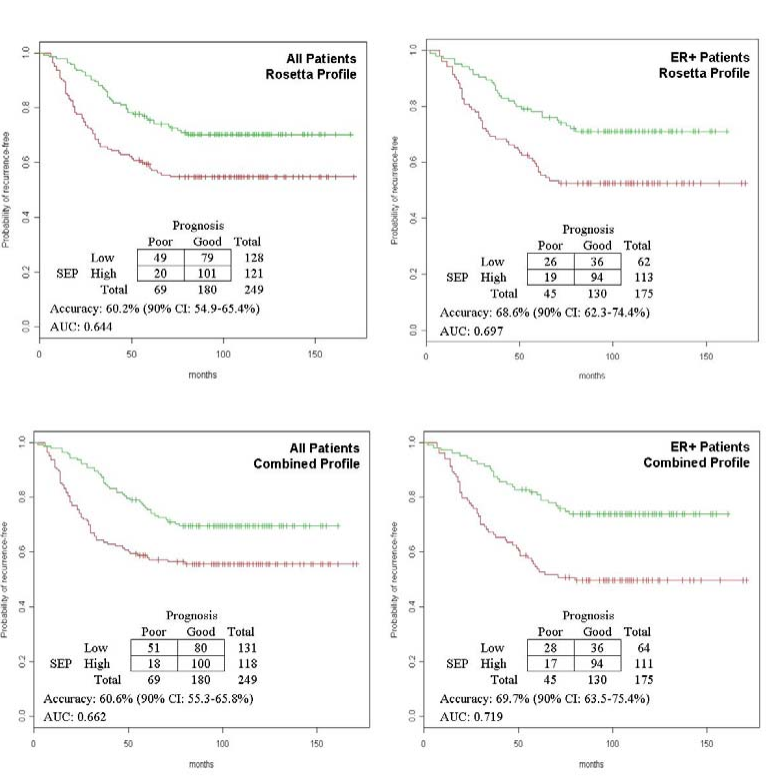
**Validation of expression profiles by a third dataset**. All 286 Veridex patients or 209 ER-positive patients were classified based on two previously derived expression profiles (Rosetta and Combined). The Kaplan-Meier survival curves corresponded to half of the patients who had SEP scores higher (green) or lower (red) than the median of all 286 scores. The contingency table, accuracy, and ROC curve area listed with plot were obtained after the Veridex patients were classified according to their 3-year prognosis as training patients.

The 60-gene profiles obtained from Rosetta and the combined datasets were validated by an independent dataset generated by Wang *et al *at Veridex, LLC [25]. This dataset included 286 lymph node-negative breast cancer patients within which 209 patients were ER-positive and 106 patients developed recurrence before the end of follow-up. The source data was downloaded from Gene Expression Omnibus database (GSE2034) and reprocessed using the same steps applied to the training datasets. The data was generated on Affymetrix Human U133A platform, which included 51 genes of both profiles. These genes and their weight were used to calculate SEP scores [see Additional file 4]. Due to the lack of common reference samples and the difference in array platforms, scores of the validating patients could not be directly compared to those of the training patients. Consequently, patients were conservatively separated into two equal-sized groups using the median score as threshold. The predictability of SEP-based classification was evaluated by Cox proportional hazards analysis (Table 6) and Kaplan-Meier survival analysis (Fig. 5). When all Veridex patients were used, both profiles significantly differentiated the recurrence outcome of high score and low score groups with very similar hazard ratios. While both profiles performed better when they were applied to ER-positive patients only, the combined dataset profile further outperformed the Rosetta profile.

**Table 6:** Cox proportional hazards analysis of SEP-based classification

**Training dataset**	**Tested on all 286 patients**		**Tested on 209 ER+ patients**	
	Hazard ratio (95% CI)	p value	Hazard ratio (95% CI)	p value

Rosetta	1.85 (1.26–2.74)	0.0019	1.97 (1.25–3.10)	0.0031
Combined	1.81 (1.23–2.66)	0.0028	2.42 (1.52–3.87)	0.0002

The 60-gene profiles obtained from the training datasets were validated by all and ER-positive patients in Veridex dataset. Patients were grouped using the median SEP score as threshold.

The correlation of genes in both profiles to 3-year prognosis was also validated. In the Veridex dataset, there were 69 patients who developed recurrence within three years and 180 patients who kept recurrence-free during a follow-up of three years or longer. The rest of the 37 patients were excluded from the following analyses. Gene differential expression between two prognosis groups was tested by Wilcox RST [see Additional file 4]. Respectively 17 and 37 of 51 genes in combined dataset profile had one-sided p values less than 0.01 and 0.1. All genes except PCTK1 had the same direction of group difference as expected. In Rosetta profile, the corresponding numbers were 9 and 28 and there were 7 genes had the opposite direction of group difference as expected.

With SEP threshold equal to the median of all 286 scores, the accuracy, specificity, and sensitivity of SEP-based classification were calculated (Fig. 5). While the overall results were poorer than the results in Table 2, the combined profile always outperformed Rosetta profile. Fisher's exact test rejected the independence of 3-year prognosis on both of combined dataset profile (p = 0.0002, odds ratio = 3.08) and Rosetta profile (p = 0.0006, odds ratio = 2.82). Notably, based on both profiles, the 50 patients having the highest scores only included four poor prognosis cases (92% specificity) while the expected number was 13.9.

### Sensitivity and specificity of expression profiles

The balance between specificity and sensitivity is a major concern of gene expression profiling. Two simulation procedures were carried out to evaluate how the change of sensitivity or specificity will affect the predictability of profiles.

**Figure 6:**
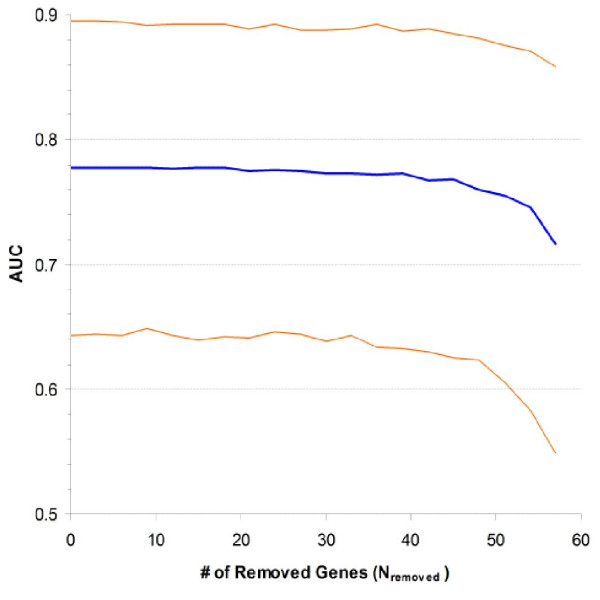
**Relationship between sensitivity and quality of expression profile**. Rosetta dataset was re-sampled for another 1,000 times. An initial 60-gene profile was obtained from each re-sampling. A stepwise reduction procedure was applied to randomly select three genes and remove them from the initial profile at each step. The consequent changing of permutation median and 90% CI of ROC curve area was presented.

The reduction procedure artificially decreased the sensitivity of expression profiles by reducing their size. The permutation re-samplings described above were applied to Rosetta dataset for another 1,000 times. The top-ranked 60 genes of each re-sampling constituted an initial profile. A stepwise procedure was applied to the initial profile by randomly removing three genes at each step until N = 0. At each reduction step, SEP of testing patients was recalculated and evaluated as a classifier of 3-year recurrence. Fig. 6 plots the changing median and 90% CI of AUC with the number of genes removed from the initial profile. The median AUC kept almost unchanged until N was 42; went down slightly when N was between 42 and 18; and dropped more quickly afterwards. The AUC had a median value of 0.716 when there were only three genes left in the profile.

**Figure 7:**
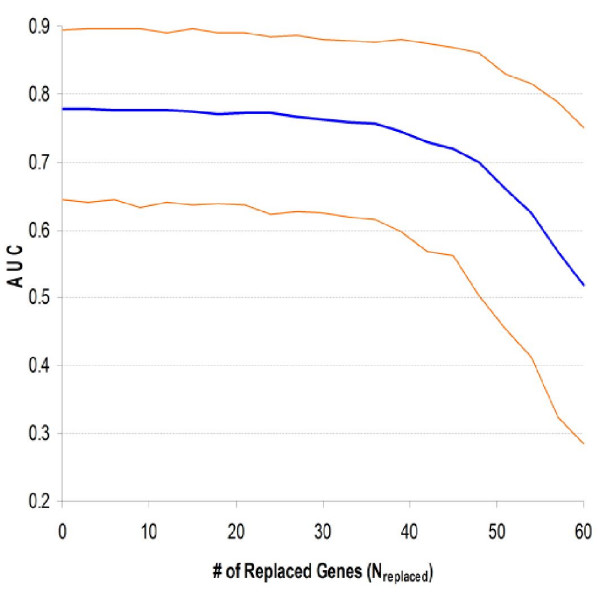
**Relationship between specificity and quality of expression profile**. After each step of the reduction procedure removed three genes (Fig. 6), these genes were replaced by three noise genes. This replacement procedure was repeated until all genes in the initial profile were false positives. The consequent changing of permutation median and 90% CI of ROC curve area was presented.

The reduction procedure artificially decreased the specificity of expression profiles by substituting top-ranked genes with false positives. Its only difference to the reduction procedure was to replace the removed genes by three other genes randomly selected from all 5,569 genes, keeping the size of expression profiles unchanged. The replacing genes inherited the weight of the replaced genes to ensure themselves as false positives. Consequently, profile specificity was gradually decreased until there were only false positives. Fig. 7 shows the changing of median and 90% CI of AUC with the number of genes replaced. The median AUC kept stable when only a few genes were replaced; dropped by about 0.02 when half genes were replaced; and fell down rapidly afterwards. Furthermore, the 90% CI was widened with the number of replaced genes. As expected, the median AUC was about 0.5 when all 60 genes were replaced.

## Discussion

A clinically valuable expression profile of general breast cancer population, if it does exist, should at least meet two requirements: it should add extra prognostic value beyond currently used indexes and it should be independent of those indexes. This study gave promising, but inconclusive, results on the first requirement. According to Table 3 and likelihood ratio test, the difference between multivariate models with and without SEP was marginally significant. However, information of important prognostic indexes, especially molecular markers such as HER2/neu and Bcl-2, were unavailable and not included in the models. Larger samples and more complete patient information are needed to draw more decisive conclusions.

This study observed the dependence of expression profiles on clinical indexes, especially ER status (Fig. 1). Such dependence was caused by confounding effect of those indexes and their unbalanced distribution between patient groups. For instance, among the 78 patients used in Fig. 1, 80% (35/44) of good prognosis patients were ER-positive while the percentage was 62% (21/34) in poor prognosis group. A partial correlation analysis was performed and successfully controlled out ER status from expression profile, but the confounding effect of other indexes remained. Although the analysis can be recursively applied to control out other indexes, the calculation of residuals by Formula (2) will introduce extra variance into the data and the expression profile obtained from partial correlation analysis failed to achieve better performance on testing patients (data not shown). As a result, this strategy is not recommended by this study unless data from much larger patient cohort is available. It was also noticed that the 60-gene profiles performed better on ER-positive patients in Veridex datasets, most likely because the majority (68%) of training patients were ER-positive. Hence, to get generally applicable profiles, confounders need to be balanced not only between prognostic subgroups but also within the complete patient cohorts.

In reality, it is difficult for single studies to accomplish large and fully balanced sample because of the limitation of resource, the large number of known and unknown confounders and their complex interaction. A more practical alternative is to diversify the clinical background and increase the overall sample size by combining multiple patient cohorts from different studies. A potential pitfall of this strategy, however, is whether independently generated datasets are combinable since the systematic bias between microarray experiments is commonly considered substantial. The current study tested the feasibility of integrated analysis by simply combining two datasets after normalizing the expression measurements within dataset. Profiles were objectively compared and the profiles of the combined dataset outperformed those of the individual datasets in most statistical analyses (Fig. 3, 5 and Table 1, 6). Furthermore, subsets of the combined dataset had better agreement on differentially expressed genes (Fig. 4), indicating that higher specificity of profiles was accomplished.

Results of this study indicated that high sensitivity of expression profile may not be necessary: median AUC reached a plateau when N was about 60 (Fig. 2); two mostly different expression profiles performed similarly in cross-validation; and more convincingly, the artificial reduction of profile sensitivity could be tolerated to an extensive level (Fig. 6). These results are consistent to the studies of Fan *et al *[13] and Ein-Dor *et al *[14], which noticed that very different profiles could significantly agree with each other and achieve equally good predictability. This observation can be explained by gene co-expression and the large number of genes correlated, directly or indirectly, to prognosis. Nevertheless, higher sensitivity may improve the robustness of profiles, which needs further investigation in future studies. On the other hand, profile specificity seems to be more critical. According to Fig. 7, performance of profiles dropped quickly when the ratio of false positives was increased. When the combined dataset profile was validated, about one-third of the genes did not have significant differential expression in Veridex patients, suggesting that specificity of this profile could be further improved. Furthermore, decreased specificity made the performance of profiles more variable (Fig. 7). For instance, while Ein-Dor *et al *noticed that there were always low-ranked genes showing quality similar to top-ranked genes, consecutive gene set often performed differently although they should have very close sensitivity and specificity.

It should be noted that genes indirectly correlated to prognosis do not fit to a profile intended to the general population because the observed correlation may be very strong in some disease subtypes, but weak or even absent in the others. The number of such 'false positives' in a profile cannot be simply estimated based on p value or false discovery rate. Instead, the ranking of genes should be derived from diversified patient cohorts, so genes directly and consistently correlated to disease outcome will have their advantage. One may question the existence of such genes and as suggested by many researchers, attempt to identify a profile for each disease subtype. However, the conclusion cannot be drawn before large-scale, cross-study screening is performed.

This study applied an atypical classification of breast cancer patients according to their 3-year prognosis. The 5-year classification, however, is commonly applied mainly for convenience, but not based on intrinsic difference of gene expression patterns between patient groups. Beside the support of Retsky model [15], 3-year classification may increase the statistical power of differential expression analysis by amplifying group difference. For instance, in the original Rosetta dataset, 1,418 of 24,481 genes were differentially expressed between 5-year prognosis groups according to RST. When 3-year classification was applied, the number of differentially expressed genes was increased to 1,759 even though the overall sample size was smaller (82 vs. 97). It was also shown that the expression profiles of 3-year prognosis were robust and successfully distinguished good and poor prognosis patients in a third dataset (Fig. 5).

SEP was demonstrated as a valuable MPI (molecular prognostic index) despite of its simple form. The parameters (gene weights) in formula (1) are estimated independent of each other, making SEP more robust than many other classifiers such as Linear Discriminate Analysis, and robustness is essential for analysis performed on independent datasets. Unlike the suggestion of Teschendorff *et al *[26], the distribution of SEP did tend to be bi-modal, or tri-modal when confounding effect was presented (Fig. 1). Although most analyses of this study dichotomized SEP scores as a conservative strategy, it is possible to apply more quantitative analysis in the future to take advantage of SEP as a continuous variable. For instance, it was demonstrated by Veridex patients that the most of highly scored patients (>90%) had good prognosis. Such high specificity, as suggested by van 't Veer *et al *[8], will help good prognosis patients avoid unnecessary radical treatments. However, we noticed that SEP scores of independent patient cohorts usually have different locations and scales. Consequently, we could only classify Veridex patients according to relative SEP values. Such a limitation of SEP or similar classifier is presumably caused by technical variations between microarray datasets, especially different array platforms. Without a common reference, the current method will not be able to classify a single testing patient before the platform and protocol of microarray experiments are standardized. To achieve the direct comparison of SEP between different patient cohorts, we suggest that all data-generating studies about the same topic should include one or more pairs of common reference samples.

## Conclusion

The current study strongly advocates the clinical value of microarray data on breast cancer prognosis and the advantage of performing expression profiling across multiple datasets. However, the generality of profiles was diminished by the confounding effect of currently used clinical indexes. A larger number of training patients with more diversified and balanced clinical background should be used by future studies to further pursue this topic.

## Methods

### Data processing

Two published microarray datasets, Rosetta [8] and Stanford [27], were used to generate gene expression profiles of breast cancer prognosis. Both datasets provided information about disease outcome and clinical indexes in addition to gene expression measurements. Breast cancer patients were classified into two prognosis groups. Patients who recurred within three years after mastectomy were classified into poor prognosis group, while those who were followed up for at least three years and kept recurrence-free at the end of follow-up were put into good prognosis group. Patients not fit to either group were excluded from this study. Consequently, Rosetta dataset included 51 good prognosis and 31 poor prognosis patients and Stanford dataset included 25 and 37 patients respectively. Microarray sequence features were mapped to NCBI Unigene clusters [28], and redundant clusters were condensed by averaging expression measurements. Totally 5,569 clusters were presented in both datasets. Only these genes were examined in this study. Sample/reference ratios were log_10_-transformed, followed by normalizing each patient to median equal zero and standard deviation equal to one. To make the same genes comparable to each other between different datasets, expression measurements of each gene were also normalized to zero median and one standard deviation separately in each dataset [see Additional file 1].

### SEP: Score for Expression Profile

A designed variable, Score for Expression Profile (SEP), was used as a weighted linear summation of gene expression profile. Given an expression profile including N genes, SEP score of each patient was calculated as:

SEP = ∑^N^[w_i_(X_i _- E_i_)]

In formula (1), w_i _was the weight of the *i*th gene, a parameter empirically estimated based on training data. When a statistical test was used to evaluate differential gene expression, the resultant test statistic or its transformation, such as correlation coefficient, Z score, or log_10_-transformed p value, could be used as the value of w. For each gene in the profile, the magnitude of its w should reflect its relative weight and the sign of its w should correspond to its direction of differential expression between sample groups. X_i _was the expression level of the *i*th gene in the patient and E_i _was its expected expression level estimated from training data. When patient outcome was dichotomous, E_i _was the expression level that had equal probability to be found in either sample group and could be denoted as E (X_i _| p_+ _= p_- _= 0.5).

### Partial correlation analysis

A partial correlation analysis was used in this study to control the confounding effect of clinical indexes on gene-outcome correlation. This analysis first controlled out a confounder from expression measurements by calculating residuals as:

X_residual _= X - E (X | Controlled Variable)

In Formula (2), X was an observed expression measurement and E was the expected value of X given a specific value of the variable to be controlled, such as positive or negative ER status. Patients were classified according to the controlled variable and the E values of each gene were estimated as group means. Subsequently, the partial correlation coefficient (r') of each gene to disease outcome was calculated using the residuals.

### Statistical analyses

Statistical analyses of this study were carried out by R 2.4.1 computing language and environment [29]. The functions used for analyses were: area of ROC curve – colAUC (package: caTools); logistic regression model: lrm (package: Design); likelihood ratio test: lrtest (package: lrtest); false discovery rate: qvalue (package: qvalue); survival analysis – survfit (package: survival); and Cox proportional hazards analysis – cph (package: Design).

## Abbreviations

AUC: Area Under (ROC) Curve; CI: Confidence Interval; ER: Estrogen Receptor; PR: Progesterone Receptor; ROC curve: Receiver Operating Characteristic curve; RST: Rank Sum test; SEP: Score for Expression Profile

### Competing interests

The author(s) declares that there are no competing interests.

## Authors' contributions

ZZ carried out this study and drafted the manuscript. DC participated in the design and execution of statistical analysis. DAF conceived of the study, provided advice, and participated in the writing of manuscript. All authors read and approved the final manuscript.

## Supplementary Material

Additional file 1 Data analysis demo. This file includes step-by-step description of data analysis procedure used in this study. Click here for file

Additional file 2 Complete gene lists of expression profiles. This file presents the complete expression profiles derived from breast cancer microarray datasets using bootstrap procedure. In each table, 'ID' is the Unigene accession of a gene while 'Name' is its symbol. 'Count' represents how many times a gene was ranked within top-100 by bootstrapping re-samplings, and 'Weight' is the Z statistic of a gene obtained from Wilcoxon Rank Sum Test (RST) applied on the data of all patients in corresponding dataset. Table 1, 2, 3 separately give the 60-gene list derived from two independent breast cancer datasets and their combination. These lists represent gene expression profiles corresponding to 3-year recurrence outcome of breast cancer and the list in Table 3 is recommended by this study. The counts are based on 10,000 re-samplings. Click here for file

Additional file 3 Functional classification of top-ranked genes. The 60 genes top-ranked by the combined dataset [see Additional File 2] was mapped to pre-defined functional gene sets using DAVID. Table 1 lists significantly enriched gene sets with their counts of genes overlapped to given 60 genes and the p values of Fisher's exact test. Table 2 lists the overlapped genes of several key gene sets. Click here for file

Additional file 4 Validation of prognostic profiles by Veridex dataset. This file provides extra validation results obtained from Veridex dataset. SEP scores of patients were calculated with 51 genes in two expression profiles and their previous derived weights. Table 1 listed the resultant scores and follow-up and ER status data provided by the original study. Table 2a and 2b listed the correlation between each of the 51 genes in both profiles and 3-year prognosis of Veridex patients. The p values were based on one-sided Wilcoxon Rank Sum test. Click here for file
