# Supplementary material for: Integrated analysis of independent gene expression microarray datasets improves the predictability of breast cancer outcome
Source: BMC Genomics. 2007 Sep 20;8:331. doi: 10.1186/1471-2164-8-331 (PMC2064937; doi:10.1186/1471-2164-8-331)
Supplement: Additional file 1 — Data analysis demo. This file includes step-by-step description of data analysis procedure used in this study. [file 1471-2164-8-331-S1.doc]

**Demo: Data Analysis Procedure**

This demo uses a simple and artificial microarray dataset of 10 breast cancer patients to demonstrate some data analysis steps utilized in this study. Table 1 and 2 separately list the clinical data of all patients and the expression measurements of all sequences in the source dataset. The original study annotated the sequences using accessions of NCBI RefSeq database and has processes microarray images to generate a 2-dimensional matrix of gene expression data. The expression profiling procedure used in this study would start with the categorization of sample patients into prognosis groups.

Table 1 Clinical Information of Sample Patients

| **Sample ID** | **Recurrence** | **Follow-up(yr)** | **ER Status** | **Tumor Size** | **Grade** | **Age(yr)** |
| --- | --- | --- | --- | --- | --- | --- |
| **p_1** | 1 | 2.53 | 1 | 2 | 2 | 43 |
| **p_2** | 0 | 6.44 | 1 | 2 | 1 | 44 |
| **p_3** | 1 | 1.66 | 0 | 2 | 3 | 41 |
| **p_4** | 1 | 1.30 | 1 | 2 | 3 | 41 |
| **p_5** | 0 | 11.98 | 0 | 2 | 3 | 48 |
| **p_6** | 1 | 1.16 | 1 | 1 | 2 | 49 |
| **p_7** | 0 | 10.14 | 0 | 2 | 1 | 46 |
| **p_8** | 0 | 8.80 | 0 | 2 | 3 | 48 |
| **p_9** | 0 | 1.29 | 1 | 1 | 3 | 48 |
| **p_10** | 1 | 6.64 | 1 | 1 | 2 | 38 |

Table 2 Expression Measurements in Source Dataset

| **Sequence ID** | **p_1** | **p_2** | **p_3** | **p_4** | **p_5** | **p_6** | **p_7** | **p_8** | **p_9** | **p_10** |
| --- | --- | --- | --- | --- | --- | --- | --- | --- | --- | --- |
| NM_003000 | 0.03 | -0.09 | 0.08 | 0.08 | -0.01 | 0.09 | 0.10 | 0.00 | -0.02 | -0.08 |
| NM_003001 | 1.11 | -0.12 | 1.28 | 0.20 | 1.11 | -0.04 | 1.07 | -0.13 | 1.02 | -0.14 |
| NM_003002 | -0.26 | -0.17 | -0.35 | -0.26 | -0.29 | 0.03 | -0.06 | 0.12 | -0.27 | -0.13 |
| NM_003003 | -0.70 | -0.04 | -0.73 | -0.05 | -0.71 | -0.03 | -0.63 | -0.01 | -0.69 | 0.03 |
| NM_003004 | 0.82 | 0.25 | 0.60 | -0.08 | 0.78 | 0.16 | 0.63 | -0.23 | 0.65 | 0.07 |
| NM_003005 | -0.89 | 0.01 | -0.81 | 0.05 | -0.80 | 0.01 | -0.60 | -0.02 | -0.77 | 0.13 |
| NM_003006 | -0.78 | -0.01 | -0.71 | 0.13 | -0.86 | -0.09 | -0.70 | -0.01 | -0.76 | -0.03 |
| NM_003007 | -1.23 | 0.25 | -1.09 | 0.23 | -1.23 | 0.05 | -1.10 | 0.04 | -1.16 | 0.45 |
| NM_003033 | -1.29 | 0.25 | -1.11 | 0.26 | -1.34 | -0.01 | -1.16 | 0.08 | -1.19 | 0.44 |
| NM_198139 | 1.09 | -0.15 | 1.06 | -0.18 | 1.21 | 0.00 | 1.16 | -0.07 | 0.95 | -0.34 |

A.1 Categorization of Sample Patients

Breast cancer patients are categorized into prognosis groups based on their follow-up data. Patients who had observed recurrence within three years after diagnosis are classified into poor prognosis group (p_1, p_3, p_4, and p_6). Patients who were followed up for at least three year and had no observed recurrence are classified into good prognosis group (p_2, p_5, p_7, and p_8). The follow-up of p_9 was too short and the recurrence of p_10 happened too late. These two patients cannot be put into either group and will be excluded from all the following steps.

A.2 Mapping Sequences to Unigene Clusters

All sequences are mapped to Unigene clusters. File containing the mapping information between RefSeq and Unigene is available at NCBI website. Both of sequence NM_003007 and NM_198139 are mapped to cluster Hs.1968 (SEMG1), so the expression measurements of these two sequences are averaged for each patient to get rid of redundancy. Sequence NM_003303 cannot be mapped to any Unigene cluster, so it is removed from the dataset. Table 3 gives the gene expression data of entire dataset after this step.

Table 3 Contents of Dataset after Sample and Sequence Filtering

| **Sequence ID** | **Gene Name** | **p_1** | **p_2** | **p_3** | **p_4** | **p_5** | ***p_6*** | **p_7** | ***p_8*** |
| --- | --- | --- | --- | --- | --- | --- | --- | --- | --- |
| Hs.1968 | SEMG1 | -0.07 | 0.05 | -0.02 | 0.03 | -0.01 | 0.02 | 0.03 | -0.02 |
| Hs.356270 | SDHD | -0.26 | -0.17 | -0.35 | -0.26 | -0.29 | 0.03 | -0.06 | 0.12 |
| Hs.444472 | SDHC | 1.11 | -0.12 | 1.28 | 0.20 | 1.11 | -0.04 | 1.07 | -0.13 |
| Hs.464184 | SEC14L1 | -0.70 | -0.04 | -0.73 | -0.05 | -0.71 | -0.03 | -0.63 | -0.01 |
| Hs.465924 | SDHB | 0.03 | -0.09 | 0.08 | 0.08 | -0.01 | 0.09 | 0.10 | 0.00 |
| Hs.506670 | SELPLG | -0.78 | -0.01 | -0.71 | 0.13 | -0.86 | -0.09 | -0.70 | -0.01 |
| Hs.546296 | SECTM1 | 0.82 | 0.25 | 0.60 | -0.08 | 0.78 | 0.16 | 0.63 | -0.23 |
| Hs.73800 | SELP | -0.89 | 0.01 | -0.81 | 0.05 | -0.80 | 0.01 | -0.60 | -0.02 |

A.3 Pre-processing of Expression Measurements

Source data are downloaded from paper websites and only genes overlapped by two training datasets are used. Fold changes of 2-channel data and summarized expression measurements of 1-channel data are log_10_-transformed, followed by two normalization steps. First, the gene expression values of each patient are subtracted by their median and then divided by their standard deviation. This step makes samples comparable to each other with the same location and similar scale. Second, in each dataset, the expression values of each gene are subtracted by their median and then divided by their standard deviation. This step brings the same genes in different datasets to the location and similar scale so they can be combined for testing differential expression. The resultant normalized data matrix is given in Table 4.

Table 4 Normalized Expression Measurements

| **Sequence ID** | **Gene Name** | **p_1** | **p_2** | **p_3** | **p_4** | **p_5** | **p_6** | **p_7** | **p_8** |
| --- | --- | --- | --- | --- | --- | --- | --- | --- | --- |
| Hs.1968 | SEMG1 | 0.14 | 2.34 | 0.61 | -0.83 | 0.45 | -0.18 | -0.14 | -0.72 |
| Hs.356270 | SDHD | 0.03 | -0.97 | -0.07 | -1.96 | -0.03 | 0.35 | 0.09 | 1.49 |
| Hs.444472 | SDHC | 0.23 | -1.62 | 0.44 | -0.22 | 0.23 | -1.60 | 0.22 | -1.91 |
| Hs.464184 | SEC14L1 | -0.14 | 1.62 | -0.18 | 0.14 | -0.25 | 0.30 | -0.84 | 2.09 |
| Hs.465924 | SDHB | 0.09 | -1.85 | 0.34 | 0.13 | -0.09 | 1.84 | -0.12 | -0.21 |
| Hs.506670 | SELPLG | -0.09 | 1.28 | 0.09 | 2.02 | -0.28 | -0.81 | -0.44 | 1.18 |
| Hs.546296 | SECTM1 | 0.11 | 0.67 | -0.07 | -1.40 | 0.07 | 0.47 | -0.11 | -2.27 |
| Hs.73800 | SELP | -0.99 | 1.38 | -0.74 | 1.00 | -0.82 | 0.74 | -0.88 | 0.77 |

A.4 Re-sampling of Patients

Permutation strategy repeatedly re-samples patients to generate training and testing subgroups. The following steps will be applied to one of such re-samplings. The results obtained from all re-samplings will be summarized to give unbiased estimation of test statistics. It is assumed that patient p_1, p_2, p_3, p_4, p_5, and p_7 are assigned to the training subgroup, leaving p_6 and p_8 in the testing subgroup.

A.5 Correlation Analysis

The Pearson correlation coefficient (r) of each gene to recurrence outcome is calculated with data of all training patients. For example, r of sequence Hs.1968 (SEMG1) is calculated with {1, 0, 1, 1, 0, 0} and {0.14, 2.34, 0.61, -0.83, 0.45, -0.14}, and the result equals to -0.47. Resultant correlation coefficients of all genes are given in Table 5. Genes are also ranked according to the magnitude of their r, from the highest to the lowest. Coefficient r can be transformed to t statistic using formula: t = r * ((n – 2) / (1 – r^2^))^1/2^, where n is sample size.

Table 5 Results of statistical tests on gene-recurrence correlation

| **Sequence ID** | **Gene Name** | **Pearson Correlation** | | **Partial Correlation** | | **Rank Sum Test** | |
| --- | --- | --- | --- | --- | --- | --- | --- |
|  |  | **r** | **rank** | **r'** | **rank** | **Z** | **rank** |
| Hs.1968 | SEMG1 | -0.47 | 7 | -0.51 | 3 | 0.65 | 5.5 |
| Hs.356270 | SDHD | -0.24 | 8 | -0.04 | 8 | 0.65 | 5.5 |
| Hs.444472 | SDHC | -0.72 | 3 | 0.73 | 2 | -0.87 | 3 |
| Hs.464184 | SEC14L1 | -0.51 | 6 | -0.48 | 5 | -0.65 | 5.5 |
| Hs.465924 | SDHB | 0.95 | 1 | 0.80 | 1 | -1.96 | 1 |
| Hs.506670 | SELPLG | 0.64 | 4 | 0.05 | 7 | -1.09 | 2 |
| Hs.546296 | SECTM1 | -0.59 | 5 | -0.49 | 4 | 0.65 | 5.5 |
| Hs.73800 | SELP | 0.92 | 2 | -0.38 | 6 | 0.22 | 8 |

A.6 Partial Correlation Analysis

ER status is the controlled variable in this demo. The following description uses sequence Hs.1968 as an example to demonstrate the process of controlling ER status from expression data.

1. Training patients are classified based on their ER status. ER-positive group includes patient p_1, p_2, and p_4 and ER-negative group includes patient p_3, p_5, and p_7.
2. Average expression level of Hs.1968 in ER-positive and –negative patients is separately calculated. The values are considered as conditional expected expression (E) of gene Hs.1968 in all patients.
   - Mean^+^ {0.14, 2.34, -0.83} = 0.55
   - Mean^−^ {0.61, 0.45, -0.14} = 0.31
3. The residuals are calculated by subtracting expression measurements in Table 4 with corresponding E values. In the case of Hs.1968, the residuals of all eight patients are:
   - {-0.41, 1.79, 0.30, -1.38, 0.14, -0.73, -0.45, -1.03}

After all expression measurements are transformed to residuals, the partial correlation coefficient (r’) between each gene and the recurrence outcome is calculated with the residuals of training patients using the same formula of Pearson correlation. Table 5 gives the r’ of each gene and the corresponding rank. The r and r’ values of some genes, such as Hs.444472, are dramatically different.

A.7 Wilcoxon Rank Sum Test (RST)

RST is performed on training data of each gene to calculate a Z statistic. The following description uses sequence Hs.1968 as an example to demonstrate the process of RST.

1. Training patients are put into two groups of opposite recurrence outcome. Group 1 includes patient p_1, p_3 and p_4, and group 2 includes patient p_2, p_5, and p_7. Size of both groups is three.
2. Expression measurements of gene Hs.1968 in all training patients are transformed to ranks. So, given data points {0.14, 2.34, 0.61, -0.83, 0.45, -0.14}, corresponding ranks will be {4, 1, 2, 6, 3, 5}. If there are equal data points, their ranks will be averaged.
3. Parameter W_1_ is calculated as the summation of ranks assigned to group 1:
   - W_1_ = Σ ranks_group1_ = rankp_1 + rankp_3 + rankp_4 = 4 + 2 + 6 = 12
4. Parameter U_1_ is calculated with W_1_ and the size of group1:
   - U_1_ = W_1_ − N_1_ (N_1_ + 1) / 2 = 12 – 3 (3 + 1) / 2 = 6
5. Mean is calculated as:
   - Mean = (N_1_ + N_2_) / 2 = (3 + 3) / 2 = 4.5
6. Variance is calculated as:
   - Variance = N_1_ N_2_ (N_1_ + N_2_ + 1) / 12 = 3 * 3 * (3 + 3 + 1) / 12 = 5.25
7. Z statistic is calculated as:
   - Z = (U – Mean) / Variance^1/2^ = (6 – 4.5) / 5.25^1/2^ = 0.655

Table 5 also gives the Z statistic of each gene and the corresponding ranks. The ranks of genes having equal Z statistics are averaged.

A.8 Calculation of SEP Score

In this demo, reporter genes are selected based on RST results. The number of reporters (N) is arbitrarily set to two. Therefore, top-ranked sequence Hs.465924 and Hs.506670 are selected as reporters and their weights are respectively -1.96 and -1.09. The SEP score of each patient is calculated with the following steps, and the intermediate results and final SEP scores are given in Table 6.

1. The expected expression level (E) of each reporter gene is calculated by averaging the expression measurement of each reporter in all training patients. In the case of Hs.465924, its value of E is calculated as:
   - Mean_Hs.465924_ {0.09, -1.85, 0.34, 0.13, -0.09, -0.12} = -0.25
2. The difference between the observed and the expected expression levels of each reporter gene is calculated and then weighted by the RST Z statistic of the gene.
3. The resultant values obtained from the last step are summed up to generate a SEP score for each patient.

The SEP scores of patients can be applied to other statistical analysis to evaluate expression profiles or the strategy used to generate them. For example, if cutoff of SEP is set as 0, both testing patient p_6 and p_8 will be classified into poor prognosis group. According to actual observation of recurrence outcome, p_6 is correctly classified, but p_8 is not, giving a classification accuracy of 50%.

Table 6 Step-by-step calculation of SEP scores

| **Patient** | **Hs.465924 (E = -0.25, W = -1.96)** | | | **Hs.506670 (E = 0.43, W = -1.09)** | | | **SEP** |
| --- | --- | --- | --- | --- | --- | --- | --- |
|  | **X** | **X - E** | **W (X - E)** | **X** | **X - E** | **W (X - E)** |  |
| **p_1** | 0.09 | 0.34 | **-0.67** | -0.09 | -0.52 | **0.57** | ***-0.10*** |
| **p_2** | -1.85 | -1.60 | **3.14** | 1.28 | 0.85 | **-0.93** | ***2.21*** |
| **p_3** | 0.34 | 0.59 | **-1.16** | 0.09 | -0.34 | **0.37** | ***-0.79*** |
| **p_4** | 0.13 | 0.38 | **-0.74** | 2.02 | 1.59 | **-1.73** | ***-2.47*** |
| **p_5** | -0.09 | 0.16 | **-0.31** | -0.28 | -0.71 | **0.77** | ***0.46*** |
| ***p_6*** | 1.84 | 2.09 | **-4.10** | -0.81 | 0.38 | **-0.41** | ***-4.51*** |
| **p_7** | -0.12 | 0.13 | **-0.25** | -0.44 | -0.01 | **0.01** | ***-0.24*** |
| ***p_8*** | -0.21 | 0.04 | **-0.08** | 1.18 | 0.75 | **-0.82** | ***-0.90*** |
