# Supplementary material for: Integrated analysis of independent gene expression microarray datasets improves the predictability of breast cancer outcome
Source: BMC Genomics. 2007 Sep 20;8:331. doi: 10.1186/1471-2164-8-331 (PMC2064937; doi:10.1186/1471-2164-8-331)
Supplement: Additional file 2 — Complete gene lists of expression profiles. This file presents the complete expression profiles derived from breast cancer microarray datasets using bootstrap procedure. In each table, 'ID' is the Unigene accession of a gene while 'Name' is its symbol. 'Count' represents how many times a gene was ranked within top-100 by bootstrapping re-samplings, and 'Weight' is the Z statistic of a gene obtained from Wilcoxon Rank Sum Test (RST) applied on the data of all patients in corresponding dataset. Table 1, 2, 3 separately give the 60-gene list derived from two independent breast cancer datasets and their combination. These lists represent gene expression profiles corresponding to 3-year recurrence outcome of breast cancer and the list in Table 3 is recommended by this study. The counts are based on 10,000 re-samplings. [file 1471-2164-8-331-S2.doc]

**Complete Gene Lists of Expression Profiles**

This file presents the complete expression profiles derived from different breast cancer microarray datasets. In each table, ‘ID’ is the Unigene accession of a gene while ‘Name’ is its symbol. ‘Count’ represents how many times a gene was ranked within top-100 by re-samplings, and ‘Weight’ is the Z statistic of a gene obtained from Wilcoxon Rank Sum Test (RST) applied on the data of all patients in corresponding dataset. Table 1-3 separately give the 60-gene list derived from two independent breast cancer datasets and their combination. These lists represent gene expression profiles corresponding to 3-year recurrence outcome of breast cancer and the list in Table 3 is recommended by this study. The counts are based on 10,000 re-samplings.

Table 1 The Reporter List Derived from Rosetta Breast Dataset

| **ID** | **Name** | **Count** | **Weight** |
| --- | --- | --- | --- |
| Hs.523468 | SCUBE2 | 9991 | 6.57 |
| Hs.445000 | PTGER3 | 9463 | 5.51 |
| Hs.148767 | RQCD1 | 9432 | -5.4 |
| Hs.352962 | LOC285086 | 9248 | -5.09 |
| Hs.496068 | PCTK1 | 8768 | -5.12 |
| Hs.520974 | YWHAG | 8743 | -5.05 |
| Hs.9589 | UBQLN1 | 8723 | -5.15 |
| Hs.433512 | ACTR3 | 8537 | -4.74 |
| Hs.20013 | SYF2 | 8213 | 4.66 |
| Hs.268787 | ARIH1 | 8119 | -4.7 |
| Hs.84113 | CDKN3 | 8114 | -4.64 |
| Hs.514527 | BIRC5 | 8079 | -4.55 |
| Hs.439200 | KIAA0090 | 8012 | 4.7 |
| Hs.2006 | GSTM3 | 7915 | 4.55 |
| Hs.380164 | KRTAP4-7 | 7855 | -4.72 |
| Hs.30743 | PRAME | 7646 | -4.68 |
| Hs.429 | ATP5G3 | 7590 | -4.52 |
| Hs.547509 | SMARCE1 | 7577 | 4.62 |
| Hs.55028 | CENPN | 7396 | -4.35 |
| Hs.482233 | DEPDC1B | 7038 | -4.25 |
| Hs.473648 | GART | 6862 | -4.39 |
| Hs.521012 | FLJ21062 | 6852 | 4.29 |
| Hs.567410 | PSMD14 | 6549 | -4.2 |
| Hs.469649 | BUB1 | 6547 | -4.2 |
| Hs.35096 |  | 6383 | 4.14 |
| Hs.495728 | PIR | 6144 | -4.12 |
| Hs.79353 | TFDP1 | 6035 | -4.11 |
| Hs.155204 | ZNF174 | 5950 | 4.12 |
| Hs.591190 | PEX12 | 5771 | 4.07 |
| Hs.518475 | EIF4A2 | 5679 | -4 |
| Hs.492618 | EXT1 | 5652 | -4 |
| Hs.173162 | COX4NB | 5647 | -4 |
| Hs.592317 | TGFB3 | 5644 | 4.02 |
| Hs.547696 | NUP155 | 5636 | -4 |
| Hs.512963 | ALG11 | 5492 | 4 |
| Hs.128425 | C19orf29 | 5463 | 4 |
| Hs.153752 | CDC25B | 5249 | -3.92 |
| Hs.190518 | C21orf45 | 5246 | -3.92 |
| Hs.350966 | PTTG1 | 5204 | -3.85 |
| Hs.484738 | MYLIP | 5159 | 3.92 |
| Hs.408062 | KLC4 | 5108 | 3.92 |
| Hs.77448 | ALDH4A1 | 5074 | 3.85 |
| Hs.517830 | BTD | 4962 | 3.92 |
| Hs.173034 |  | 4940 | 3.85 |
| Hs.433951 | GPX4 | 4831 | 3.8 |
| Hs.390729 | ERBB4 | 4811 | 3.8 |
| Hs.518464 | PSMD2 | 4717 | -3.8 |
| Hs.491148 | PCM1 | 4652 | 3.74 |
| Hs.567267 | FANCA | 4650 | -3.8 |
| Hs.188569 | ZDHHC13 | 4647 | -3.8 |
| Hs.508716 | COL4A2 | 4646 | -3.74 |
| Hs.65758 | ITPR3 | 4548 | -3.74 |
| Hs.81934 | ACADSB | 4538 | 3.8 |
| Hs.83383 | PRDX4 | 4515 | -3.7 |
| Hs.532803 | HN1 | 4382 | -3.7 |
| Hs.436187 | TRIP13 | 4343 | -3.74 |
| Hs.308045 | NCAPH | 4308 | -3.7 |
| Hs.113876 | WHSC1 | 4303 | -3.66 |
| Hs.153357 | PLOD3 | 4281 | -3.66 |
| Hs.576154 | LRP8 | 4211 | -3.7 |

Table 2 The Reporter List derived from Stanford Breast Dataset

| **ID** | **Name** | **Count** | **Weight** |
| --- | --- | --- | --- |
| Hs.523836 | GSTP1 | 9866 | -5.17 |
| Hs.208124 | ESR1 | 9297 | 4.33 |
| Hs.597547 |  | 9268 | -4.25 |
| Hs.58974 | CCNA2 | 8925 | -4.09 |
| Hs.211589 | PPEF1 | 8822 | 4.28 |
| Hs.584238 | GLDC | 8755 | -4.14 |
| Hs.502769 | SLC3A2 | 8719 | -4.11 |
| Hs.94865 | TEAD4 | 8599 | -4.07 |
| Hs.460468 | XPO6 | 8465 | -4.04 |
| Hs.292579 | PTDSS1 | 8305 | -3.92 |
| Hs.469649 | BUB1 | 8049 | -4 |
| Hs.371013 | JMJD2B | 8019 | 3.8 |
| Hs.150749 | BCL2 | 8003 | 3.85 |
| Hs.647069 | SLC4A2 | 7632 | -3.74 |
| Hs.568928 |  | 7585 | 3.7 |
| Hs.567352 | TXNRD1 | 7402 | -3.62 |
| Hs.224607 | SDC1 | 6652 | -3.47 |
| Hs.199487 | RERG | 6635 | 3.42 |
| Hs.178695 | MAPK13 | 6614 | -3.4 |
| Hs.267659 | VAV3 | 6421 | 3.42 |
| Hs.647036 | CLDN4 | 6149 | -3.36 |
| Hs.647051 | BCL7B | 6099 | -3.28 |
| Hs.159799 | THRAP2 | 5763 | 3.24 |
| Hs.102471 | PHACTR2 | 5627 | -3.22 |
| Hs.524134 | GATA3 | 5530 | 3.21 |
| Hs.78619 | GGH | 5439 | -3.19 |
| Hs.3416 | ADFP | 5113 | -3.1 |
| Hs.515046 | SLC39A3 | 5067 | -3.07 |
| Hs.197320 | TLE1 | 5043 | -3.08 |
| Hs.530735 | MS4A7 | 4994 | 3.08 |
| Hs.9661 | PSMB10 | 4918 | -3.05 |
| Hs.647078 | CDK5 | 4698 | -3 |
| Hs.526879 | PTPRT | 4690 | 2.98 |
| Hs.83114 | CRYZ | 4581 | 2.97 |
| Hs.69771 | CFB | 4561 | 3 |
| Hs.498661 | USP6NL | 4555 | -2.97 |
| Hs.466471 | GPI | 4524 | -2.98 |
| Hs.520026 | VARS | 4432 | -2.95 |
| Hs.368149 | CCT7 | 4333 | -2.94 |
| Hs.592095 | SLC16A5 | 4281 | -2.94 |
| Hs.91728 | EXOSC9 | 4109 | -2.89 |
| Hs.118351 | UBE3C | 4019 | -2.82 |
| Hs.471156 | ABI2 | 3993 | 2.82 |
| Hs.5719 | NCAPD2 | 3984 | -2.86 |
| Hs.150444 | CEP290 | 3979 | 2.83 |
| Hs.82963 | GNRH1 | 3971 | 2.85 |
| Hs.370392 | MYO1E | 3965 | -2.85 |
| Hs.179718 | MYBL2 | 3913 | -2.85 |
| Hs.529303 | ARPC2 | 3862 | -2.83 |
| Hs.576154 | LRP8 | 3858 | -2.85 |
| Hs.460184 | MCM4 | 3857 | -2.8 |
| Hs.483444 | CXCL14 | 3836 | 2.82 |
| Hs.183800 | RANGAP1 | 3829 | -2.8 |
| Hs.516159 | USP39 | 3826 | -2.79 |
| Hs.462341 | M-RIP | 3812 | -2.79 |
| Hs.443861 | SRPK1 | 3810 | -2.82 |
| Hs.250712 | CACNB3 | 3770 | -2.77 |
| Hs.510989 | MEIS2 | 3754 | -2.79 |
| Hs.644621 | C14orf45 | 3695 | 2.77 |
| Hs.182385 | HPN | 3685 | 2.77 |

Table 3 The Reporter List Derived from the Combination of Two Breast Datasets

| **ID** | **Name** | **Count** | **Weight** |
| --- | --- | --- | --- |
| Hs.496068 | PCTK1 | 9862 | -4.94 |
| Hs.523468 | SCUBE2 | 9732 | 5.18 |
| Hs.469649 | BUB1 | 9681 | -4.56 |
| Hs.208124 | ESR1 | 9647 | 5.23 |
| Hs.35096 |  | 9559 | 5.14 |
| Hs.520974 | YWHAG | 9426 | -4.94 |
| Hs.436187 | TRIP13 | 9360 | -4.48 |
| Hs.173162 | COX4NB | 8970 | -4.42 |
| Hs.58974 | CCNA2 | 8673 | -4.17 |
| Hs.524134 | GATA3 | 8651 | 4.93 |
| Hs.514527 | BIRC5 | 8595 | -4.6 |
| Hs.82906 | MPL | 8466 | -4.42 |
| Hs.576154 | LRP8 | 8428 | -4.76 |
| Hs.267659 | VAV3 | 8415 | 5.01 |
| Hs.78619 | GGH | 8407 | -4.29 |
| Hs.308045 | NCAPH | 8299 | -4.5 |
| Hs.188569 | ZDHHC13 | 8176 | -4.43 |
| Hs.12272 | BECN1 | 8158 | 4.92 |
| Hs.3416 | ADFP | 8043 | -4.45 |
| Hs.292579 | PTDSS1 | 8035 | -4.36 |
| Hs.83383 | PRDX4 | 7839 | -4.36 |
| Hs.79353 | TFDP1 | 7821 | -4.32 |
| Hs.371013 | JMJD2B | 7750 | 4.94 |
| Hs.153752 | CDC25B | 7690 | -4.47 |
| Hs.9589 | UBQLN1 | 7634 | -3.94 |
| Hs.445000 | PTGER3 | 7542 | 4.79 |
| Hs.2006 | GSTM3 | 7365 | 4.62 |
| Hs.190518 | C21orf45 | 7178 | -4.69 |
| Hs.482233 | DEPDC1B | 7061 | -3.9 |
| Hs.150749 | BCL2 | 7047 | 4.59 |
| Hs.12109 | CIAO1 | 6994 | -4.07 |
| Hs.5719 | NCAPD2 | 6931 | -4.1 |
| Hs.567352 | TXNRD1 | 6828 | -4.21 |
| Hs.78771 | PGK1 | 6698 | -4.14 |
| Hs.111554 | ARL4C | 6626 | -4.41 |
| Hs.65758 | ITPR3 | 6519 | -4.24 |
| Hs.81934 | ACADSB | 6417 | 4.68 |
| Hs.374378 | CKS1B | 6363 | -4.07 |
| Hs.197320 | TLE1 | 6356 | -4.3 |
| Hs.483444 | CXCL14 | 6352 | 4.37 |
| Hs.532803 | HN1 | 6339 | -4.34 |
| Hs.435326 | ACTL6A | 6336 | -3.91 |
| Hs.153357 | PLOD3 | 6083 | -3.98 |
| Hs.350966 | PTTG1 | 6024 | -3.96 |
| Hs.433512 | ACTR3 | 5987 | -4.13 |
| Hs.632299 | NUP205 | 5963 | -4.26 |
| Hs.498661 | USP6NL | 5925 | -4.16 |
| Hs.491148 | PCM1 | 5901 | 4.79 |
| Hs.584836 | ITGBL1 | 5724 | 4.38 |
| Hs.492618 | EXT1 | 5655 | -4.17 |
| Hs.524399 | TROAP | 5653 | -4.16 |
| Hs.530735 | MS4A7 | 5600 | 4.74 |
| Hs.436912 | KIFC1 | 5402 | -3.95 |
| Hs.495728 | PIR | 5357 | -3.96 |
| Hs.486401 | C6orf173 | 5350 | -3.99 |
| Hs.409065 | FEN1 | 5326 | -4.02 |
| Hs.226390 | RRM2 | 5259 | -3.99 |
| Hs.473648 | GART | 5256 | -3.88 |
| Hs.513797 | SLC7A5 | 5216 | -4.14 |
| Hs.69771 | CFB | 5168 | 4.08 |
