# Supplementary material for: Integrated analysis of independent gene expression microarray datasets improves the predictability of breast cancer outcome
Source: BMC Genomics. 2007 Sep 20;8:331. doi: 10.1186/1471-2164-8-331 (PMC2064937; doi:10.1186/1471-2164-8-331)
Supplement: Additional file 3 — Functional classification of top-ranked genes. The 60 genes top-ranked by the combined dataset [see Additional File 2] was mapped to pre-defined functional gene sets using DAVID. Table 1 lists significantly enriched gene sets with their counts of genes overlapped to given 60 genes and the p values of Fisher's exact test. Table 2 lists the overlapped genes of several key gene sets. [file 1471-2164-8-331-S3.doc]

### Functional classification of top-ranked genes

The 60 genes top-ranked by the combined dataset (see Supplemental File 2) was mapped to pre-defined functional gene sets using DAVID. Table 1 lists significantly enriched gene sets with their counts of genes overlapped to given 60 genes and the p values of Fisher Exact test. Table 2 lists the overlapped genes of several key gene sets.

Table 1 Enriched Gene Sets

| **Category** | **Term** | **Count** | **PValue** |
| --- | --- | --- | --- |
| GOTERM_BP_ALL | mitosis | 8 | 0.00001 |
| GOTERM_BP_ALL | M phase of mitotic cell cycle | 8 | 0.00001 |
| GOTERM_BP_ALL | mitotic cell cycle | 9 | 0.00001 |
| GOTERM_BP_ALL | cell cycle | 14 | 0.00008 |
| SP_PIR_KEYWORDS | cell division | 6 | 0.001 |
| SP_PIR_KEYWORDS | mitosis | 5 | 0.001 |
| GOTERM_BP_ALL | chromosome segregation | 4 | 0.001 |
| SP_PIR_KEYWORDS | cell cycle | 8 | 0.002 |
| GOTERM_BP_ALL | cell division | 6 | 0.002 |
| GOTERM_BP_ALL | regulation of progression through cell cycle | 9 | 0.004 |
| GOTERM_BP_ALL | regulation of cell cycle | 9 | 0.004 |
| GOTERM_BP_ALL | sister chromatid segregation | 3 | 0.006 |
| GOTERM_BP_ALL | mitotic sister chromatid segregation | 3 | 0.006 |
| BIND | 2885675:MYOSIN, HEAVY POLYPEPTIDE 10, NON-MUSCLE | 5 | 0.013 |
| BIND | 2878696:ACTIN, BETA | 5 | 0.016 |
| BIND | 2877939:GROWTH FACTOR RECEPTOR-BOUND PROTEIN 2 | 5 | 0.020 |
| GOTERM_BP_ALL | regulation of mitosis | 3 | 0.027 |
| BIND | 2867166:E2F TRANSCRIPTION FACTOR 5, P130-BINDING | 2 | 0.029 |
| BIND | 3029231:CDC20 CELL DIVISION CYCLE 20 HOMOLOG (S. CEREVISIAE) | 2 | 0.029 |
| MINT | 3027732:BCL2-ASSOCIATED TRANSCRIPTION FACTOR 1 | 2 | 0.032 |
| SP_PIR_KEYWORDS | dna condensation | 2 | 0.037 |
| SP_PIR_KEYWORDS | Phosphorylation | 5 | 0.038 |
| GOTERM_BP_ALL | mitotic chromosome condensation | 2 | 0.041 |
| SMART_NAME | SM00268:ACTIN | 2 | 0.044 |
| BIND | 2874445:E2F TRANSCRIPTION FACTOR 4, P107/P130-BINDING | 3 | 0.045 |
| GOTERM_BP_ALL | negative regulation of apoptosis | 4 | 0.046 |
| GOTERM_BP_ALL | negative regulation of programmed cell death | 4 | 0.046 |
| INTERPRO_NAME | IPR004000:Actin/actin-like | 2 | 0.049 |
| SP_PIR_KEYWORDS | phosphorylation | 17 | 0.050 |
| GOTERM_BP_ALL | chromosome condensation | 2 | 0.054 |
| GOTERM_BP_ALL | signal transduction | 18 | 0.056 |
| GOTERM_CC_ALL | microtubule cytoskeleton | 4 | 0.071 |
| INTERPRO_NAME | IPR012335:Thioredoxin fold | 3 | 0.076 |
| GOTERM_MF_ALL | enzyme regulator activity | 7 | 0.085 |
| MINT | 2873661:PRESENILIN 1 (ALZHEIMER DISEASE 3) | 2 | 0.094 |
| INTERPRO_NAME | IPR012336:Thioredoxin-like fold | 3 | 0.095 |

Table 2 Overlapped Genes of Selected Key Gene Sets

| **Gene_Set** | **Unigene** | **GENE** |
| --- | --- | --- |
| Cell Cycle | Hs.153752 | CDC25B |
| Cell Cycle | Hs.469649 | BUB1 |
| Cell Cycle | Hs.492618 | EXT1 |
| Cell Cycle | Hs.150749 | BCL2 |
| Cell Cycle | Hs.208124 | ESR1 |
| Cell Cycle | Hs.350966 | PTTG1 |
| Cell Cycle | Hs.496068 | PCTK1 |
| Cell Cycle | Hs.308045 | NCAPH |
| Cell Cycle | Hs.374378 | CKS1B |
| Cell Cycle | Hs.5719 | NCAPD2 |
| Cell Cycle | Hs.79353 | TFDP1 |
| Cell Cycle | Hs.520974 | YWHAG |
| Cell Cycle | Hs.436912 | KIFC1 |
| Cell Cycle | Hs.514527 | BIRC5 |
| E2F TRANSCRIPTION FACTOR 4, P107/P130-BINDING | Hs.79353 | TFDP1 |
| E2F TRANSCRIPTION FACTOR 4, P107/P130-BINDING | Hs.486401 | C6orf173 |
| E2F TRANSCRIPTION FACTOR 4, P107/P130-BINDING | Hs.514527 | BIRC5 |
| Enzyme Regulatory Activity | Hs.267659 | VAV3 |
| Enzyme Regulatory Activity | Hs.374378 | CKS1B |
| Enzyme Regulatory Activity | Hs.498661 | USP6NL |
| Enzyme Regulatory Activity | Hs.520974 | YWHAG |
| Enzyme Regulatory Activity | Hs.514527 | BIRC5 |
| Enzyme Regulatory Activity | Hs.208124 | ESR1 |
| Enzyme Regulatory Activity | Hs.350966 | PTTG1 |
| microtubule cytoskeleton | Hs.491148 | PCM1 |
| microtubule cytoskeleton | Hs.469649 | BUB1 |
| microtubule cytoskeleton | Hs.436912 | KIFC1 |
| microtubule cytoskeleton | Hs.514527 | BIRC5 |
| MYOSIN, HEAVY POLYPEPTIDE 10, NON-MUSCLE | Hs.65758 | ITPR3 |
| MYOSIN, HEAVY POLYPEPTIDE 10, NON-MUSCLE | Hs.267659 | VAV3 |
| MYOSIN, HEAVY POLYPEPTIDE 10, NON-MUSCLE | Hs.498661 | USP6NL |
| MYOSIN, HEAVY POLYPEPTIDE 10, NON-MUSCLE | Hs.2006 | GSTM3 |
| MYOSIN, HEAVY POLYPEPTIDE 10, NON-MUSCLE | Hs.433512 | ACTR3 |
| negative regulation of apoptosis | Hs.150749 | BCL2 |
| negative regulation of apoptosis | Hs.12272 | BECN1 |
| negative regulation of apoptosis | Hs.520974 | YWHAG |
| negative regulation of apoptosis | Hs.514527 | BIRC5 |
| Signal Transduction | Hs.111554 | ARL4C |
| Signal Transduction | Hs.445000 | PTGER3 |
| Signal Transduction | Hs.584836 | ITGBL1 |
| Signal Transduction | Hs.492618 | EXT1 |
| Signal Transduction | Hs.482233 | DEPDC1B |
| Signal Transduction | Hs.483444 | CXCL14 |
| Signal Transduction | Hs.208124 | ESR1 |
| Signal Transduction | Hs.82906 | MPL |
| Signal Transduction | Hs.83383 | PRDX4 |
| Signal Transduction | Hs.65758 | ITPR3 |
| Signal Transduction | Hs.197320 | TLE1 |
| Signal Transduction | Hs.530735 | MS4A7 |
| Signal Transduction | Hs.267659 | VAV3 |
| Signal Transduction | Hs.520974 | YWHAG |
| Signal Transduction | Hs.567352 | TXNRD1 |
| Signal Transduction | Hs.188569 | ZDHHC13 |
| Signal Transduction | Hs.435326 | ACTL6A |
| Signal Transduction | Hs.409065 | FEN1 |
