# Supplementary material for: Integrated analysis of independent gene expression microarray datasets improves the predictability of breast cancer outcome
Source: BMC Genomics. 2007 Sep 20;8:331. doi: 10.1186/1471-2164-8-331 (PMC2064937; doi:10.1186/1471-2164-8-331)
Supplement: Additional file 4 — Validation of prognostic profiles by Veridex dataset. This file provides extra validation results obtained from Veridex dataset. SEP scores of patients were calculated with 51 genes in two expression profiles and their previous derived weights. Table 1 listed the resultant scores and follow-up and ER status data provided by the original study. Table 2a and 2b listed the correlation between each of the 51 genes in both profiles and 3-year prognosis of Veridex patients. The p values were based on one-sided Wilcoxon Rank Sum test. [file 1471-2164-8-331-S4.doc]

### Validation of prognostic profiles by Veridex dataset

This file provides extra validation results obtained from Veridex dataset. SEP scores of patients were calculated with 51 genes in two expression profiles and their previous derived weights. Table 1 listed the resultant scores and follow-up and ER status data provided by the original study. Table 2a and 2b listed the correlation between each of the 51 genes in both profiles and 3-year prognosis of Veridex patients. The p values were based on one-sided Wilcoxon Rank Sum test.

Table 1 SEP scores of Veridex patients

| Sample  ID | Array  ID | ER  Status | Time  (month) | Relapse  (true=1) | SEP  (combined) | SEP  (Rosetta) |
| --- | --- | --- | --- | --- | --- | --- |
| 3 | GSM36793 | ER- | 101 | 0 | -22.9 | 8.4 |
| 5 | GSM36796 | ER+ | 118 | 0 | 17.1 | 21.6 |
| 6 | GSM36797 | ER- | 9 | 1 | -194.6 | -152.1 |
| 7 | GSM36798 | ER- | 106 | 0 | -116.1 | -115.4 |
| 8 | GSM36800 | ER- | 37 | 1 | 21.7 | 34.2 |
| 9 | GSM36801 | ER+ | 125 | 0 | 2.5 | 26.7 |
| 11 | GSM36834 | ER+ | 109 | 0 | -163.8 | -122.0 |
| 14 | GSM36835 | ER- | 14 | 1 | -176.8 | -151.3 |
| 15 | GSM36836 | ER+ | 99 | 0 | 64.2 | 100.9 |
| 17 | GSM36837 | ER+ | 137 | 0 | 10.6 | 32.7 |
| 18 | GSM36838 | ER+ | 34 | 1 | 34.9 | 30.0 |
| 19 | GSM36839 | ER+ | 32 | 1 | 156.3 | 132.3 |
| 20 | GSM36855 | ER- | 128 | 0 | -137.5 | -96.9 |
| 21 | GSM36858 | ER+ | 14 | 1 | -43.8 | -20.5 |
| 22 | GSM36859 | ER+ | 130 | 0 | 117.0 | 82.0 |
| 27 | GSM36860 | ER+ | 30 | 1 | -58.5 | -27.9 |
| 28 | GSM36861 | ER+ | 155 | 0 | 156.2 | 104.2 |
| 29 | GSM36862 | ER- | 25 | 1 | -169.8 | -119.3 |
| 31 | GSM36870 | ER+ | 30 | 1 | -34.2 | -2.0 |
| 32 | GSM36871 | ER+ | 84 | 0 | 141.6 | 134.1 |
| 33 | GSM36872 | ER+ | 7 | 1 | -107.8 | -77.4 |
| 34 | GSM36873 | ER+ | 100 | 0 | -13.1 | 5.1 |
| 35 | GSM36874 | ER+ | 30 | 1 | 10.6 | 21.6 |
| 37 | GSM36875 | ER- | 7 | 1 | -187.6 | -177.5 |
| 38 | GSM36876 | ER- | 133 | 0 | -199.6 | -172.8 |
| 39 | GSM36877 | ER+ | 43 | 1 | -64.0 | -79.0 |
| 40 | GSM36896 | ER+ | 102 | 0 | 94.5 | 96.4 |
| 41 | GSM36897 | ER+ | 25 | 1 | 74.9 | 47.1 |
| 44 | GSM36899 | ER+ | 169 | 0 | -49.3 | 19.7 |
| 46 | GSM36900 | ER+ | 109 | 0 | -142.3 | -90.9 |
| 48 | GSM36901 | ER+ | 101 | 0 | -215.0 | -166.1 |
| 49 | GSM36902 | ER+ | 28 | 1 | 19.3 | 36.5 |
| 50 | GSM36916 | ER+ | 125 | 0 | 123.7 | 113.2 |
| 54 | GSM36917 | ER+ | 144 | 0 | 126.6 | 102.1 |
| 55 | GSM36918 | ER- | 11 | 1 | -39.6 | -16.5 |
| 56 | GSM36919 | ER+ | 153 | 0 | 90.1 | 74.8 |
| 57 | GSM36920 | ER+ | 20 | 1 | 11.5 | 20.3 |
| 61 | GSM36937 | ER- | 11 | 1 | -194.8 | -174.3 |
| 62 | GSM36938 | ER+ | 108 | 0 | 59.5 | 81.5 |
| 63 | GSM36939 | ER+ | 14 | 1 | -68.5 | -22.2 |
| 64 | GSM36940 | ER- | 134 | 0 | -189.1 | -115.7 |
| 65 | GSM36941 | ER- | 9 | 1 | -208.7 | -162.1 |
| 66 | GSM36942 | ER+ | 152 | 0 | 67.4 | 60.5 |
| 67 | GSM36943 | ER+ | 19 | 1 | -0.9 | -34.1 |
| 68 | GSM36944 | ER+ | 112 | 0 | 104.5 | 88.3 |
| 70 | GSM36962 | ER+ | 114 | 0 | 168.1 | 152.5 |
| 71 | GSM36964 | ER- | 12 | 1 | -87.8 | -41.5 |
| 72 | GSM36965 | ER+ | 134 | 0 | 65.3 | 52.3 |
| 74 | GSM36966 | ER- | 90 | 0 | -188.6 | -120.3 |
| 75 | GSM36967 | ER+ | 15 | 1 | 11.7 | 9.1 |
| 78 | GSM36968 | ER- | 152 | 0 | -148.0 | -42.3 |
| 79 | GSM36969 | ER- | 32 | 1 | -228.0 | -195.6 |
| 80 | GSM36987 | ER+ | 97 | 0 | 18.5 | -8.1 |
| 81 | GSM36989 | ER+ | 11 | 1 | 4.7 | 12.4 |
| 82 | GSM36990 | ER+ | 143 | 0 | 125.7 | 107.8 |
| 84 | GSM36991 | ER- | 114 | 0 | -209.9 | -178.0 |
| 86 | GSM36992 | ER+ | 104 | 0 | -73.3 | -11.4 |
| 88 | GSM36993 | ER+ | 98 | 0 | 128.4 | 133.1 |
| 89 | GSM36994 | ER+ | 2 | 1 | 120.6 | 108.8 |
| 90 | GSM37017 | ER- | 108 | 0 | -174.4 | -140.0 |
| 91 | GSM37018 | ER+ | 20 | 1 | -45.6 | -51.5 |
| 92 | GSM37019 | ER+ | 146 | 0 | 54.2 | 43.7 |
| 93 | GSM37020 | ER- | 48 | 1 | 10.5 | 25.5 |
| 94 | GSM37021 | ER- | 109 | 0 | -241.9 | -174.4 |
| 96 | GSM37022 | ER- | 30 | 1 | -149.0 | -98.7 |
| 98 | GSM37023 | ER- | 14 | 1 | -72.5 | -33.5 |
| 99 | GSM37025 | ER+ | 107 | 0 | 63.5 | 54.3 |
| 100 | GSM36879 | ER- | 39 | 1 | -33.0 | 1.6 |
| 103 | GSM36880 | ER+ | 97 | 0 | 30.4 | 45.1 |
| 104 | GSM36881 | ER+ | 11 | 1 | -42.7 | -22.8 |
| 105 | GSM36882 | ER+ | 99 | 0 | 68.7 | 79.6 |
| 106 | GSM36885 | ER+ | 40 | 1 | 132.9 | 102.1 |
| 107 | GSM36886 | ER- | 94 | 0 | -46.9 | -56.9 |
| 108 | GSM36888 | ER+ | 28 | 1 | -14.9 | -42.8 |
| 109 | GSM36891 | ER- | 87 | 0 | -123.5 | -105.0 |
| 110 | GSM36903 | ER+ | 8 | 1 | -38.0 | -51.3 |
| 111 | GSM36904 | ER- | 98 | 0 | 19.6 | 33.1 |
| 112 | GSM36905 | ER- | 17 | 1 | -2.1 | 31.9 |
| 113 | GSM36906 | ER- | 101 | 0 | -145.5 | -83.2 |
| 114 | GSM36907 | ER+ | 86 | 0 | 61.5 | 70.7 |
| 115 | GSM36908 | ER+ | 15 | 1 | 35.0 | 38.2 |
| 118 | GSM36909 | ER- | 95 | 0 | -15.5 | -9.0 |
| 121 | GSM36923 | ER- | 23 | 1 | -144.9 | -119.9 |
| 122 | GSM36925 | ER+ | 104 | 0 | 103.7 | 99.4 |
| 124 | GSM36927 | ER+ | 8 | 1 | -192.2 | -172.2 |
| 125 | GSM36929 | ER+ | 93 | 0 | 21.7 | 23.3 |
| 126 | GSM36931 | ER+ | 37 | 1 | -118.6 | -76.3 |
| 130 | GSM36946 | ER+ | 26 | 1 | -27.6 | -3.1 |
| 133 | GSM36948 | ER+ | 87 | 0 | -15.4 | 3.6 |
| 134 | GSM36950 | ER+ | 28 | 1 | 83.5 | 77.9 |
| 135 | GSM36953 | ER- | 84 | 0 | -76.0 | -56.2 |
| 136 | GSM36955 | ER- | 25 | 1 | -38.9 | -20.4 |
| 137 | GSM36956 | ER+ | 32 | 1 | 46.7 | 35.6 |
| 138 | GSM36957 | ER+ | 47 | 1 | 116.3 | 87.3 |
| 139 | GSM36958 | ER+ | 111 | 0 | -12.8 | 1.3 |
| 140 | GSM36971 | ER+ | 19 | 1 | 52.1 | 38.2 |
| 141 | GSM36973 | ER+ | 25 | 1 | 32.1 | 48.1 |
| 200 | GSM36921 | ER+ | 108 | 0 | 29.0 | 45.4 |
| 201 | GSM36922 | ER+ | 113 | 0 | 27.2 | 23.6 |
| 203 | GSM36924 | ER+ | 29 | 1 | -68.1 | -28.2 |
| 204 | GSM36926 | ER- | 24 | 1 | -57.9 | -17.5 |
| 205 | GSM36928 | ER+ | 23 | 1 | 61.0 | 61.0 |
| 212 | GSM36898 | ER+ | 7 | 1 | -35.2 | -14.1 |
| 213 | GSM36947 | ER+ | 16 | 1 | -90.1 | -90.9 |
| 215 | GSM36949 | ER- | 6 | 1 | -244.1 | -194.2 |
| 216 | GSM36952 | ER- | 13 | 1 | -64.8 | -70.2 |
| 217 | GSM36954 | ER+ | 19 | 1 | -136.4 | -57.6 |
| 222 | GSM36972 | ER+ | 37 | 1 | 131.6 | 70.3 |
| 223 | GSM36974 | ER+ | 23 | 1 | -61.6 | -90.4 |
| 226 | GSM36976 | ER+ | 38 | 1 | 37.9 | 69.8 |
| 230 | GSM36996 | ER+ | 24 | 1 | -31.0 | -13.2 |
| 231 | GSM36997 | ER+ | 44 | 1 | 31.5 | 10.0 |
| 233 | GSM36998 | ER+ | 5 | 1 | 104.1 | 94.5 |
| 234 | GSM36999 | ER+ | 37 | 1 | 125.6 | 80.1 |
| 235 | GSM37001 | ER+ | 33 | 1 | 16.0 | 16.3 |
| 236 | GSM37002 | ER- | 16 | 1 | -184.6 | -135.4 |
| 237 | GSM37003 | ER+ | 19 | 1 | -108.7 | -72.1 |
| 239 | GSM37004 | ER+ | 35 | 1 | 114.5 | 52.8 |
| 240 | GSM37026 | ER+ | 36 | 1 | 70.7 | 57.9 |
| 241 | GSM37027 | ER+ | 17 | 1 | 4.3 | -1.2 |
| 244 | GSM37028 | ER+ | 39 | 1 | 99.4 | 78.6 |
| 246 | GSM37029 | ER+ | 47 | 1 | -5.4 | 18.5 |
| 247 | GSM37030 | ER+ | 44 | 1 | 89.7 | 57.6 |
| 249 | GSM37031 | ER+ | 18 | 1 | -53.1 | -31.5 |
| 250 | GSM37035 | ER+ | 14 | 1 | -64.7 | 5.7 |
| 252 | GSM37036 | ER+ | 15 | 1 | 61.4 | 62.4 |
| 253 | GSM37037 | ER+ | 19 | 1 | 42.0 | 17.1 |
| 254 | GSM37039 | ER+ | 48 | 1 | 135.0 | 74.8 |
| 255 | GSM37040 | ER- | 6 | 1 | -174.3 | -142.3 |
| 258 | GSM37041 | ER+ | 9 | 1 | 68.6 | 37.0 |
| 259 | GSM37042 | ER- | 14 | 1 | -72.8 | -47.2 |
| 260 | GSM37049 | ER- | 29 | 1 | 2.1 | -10.6 |
| 261 | GSM37050 | ER- | 18 | 1 | 24.2 | -0.3 |
| 262 | GSM37051 | ER+ | 18 | 1 | -61.4 | -59.1 |
| 263 | GSM37052 | ER- | 32 | 1 | -73.2 | -63.5 |
| 264 | GSM37053 | ER- | 33 | 1 | -119.8 | -25.0 |
| 265 | GSM37054 | ER- | 66 | 0 | -82.5 | -58.4 |
| 267 | GSM37055 | ER- | 59 | 0 | -57.0 | -31.0 |
| 268 | GSM37056 | ER- | 82 | 0 | 132.8 | 116.7 |
| 270 | GSM37057 | ER+ | 59 | 0 | -1.8 | -12.9 |
| 271 | GSM37058 | ER+ | 64 | 1 | 26.8 | 16.8 |
| 272 | GSM37059 | ER+ | 83 | 0 | 151.4 | 118.5 |
| 273 | GSM37060 | ER+ | 81 | 0 | -11.9 | 17.1 |
| 275 | GSM37061 | ER- | 105 | 0 | 124.2 | 108.5 |
| 276 | GSM37062 | ER+ | 54 | 0 | -51.8 | -41.0 |
| 277 | GSM36777 | ER+ | 79 | 0 | 102.9 | 90.6 |
| 278 | GSM36778 | ER+ | 50 | 1 | -32.5 | -50.8 |
| 284 | GSM36787 | ER+ | 72 | 0 | 52.2 | 24.5 |
| 285 | GSM36789 | ER+ | 51 | 1 | 35.6 | 58.4 |
| 286 | GSM36790 | ER+ | 107 | 0 | 130.5 | 105.8 |
| 287 | GSM36791 | ER- | 79 | 0 | 106.8 | 67.8 |
| 288 | GSM36792 | ER+ | 71 | 1 | 75.4 | 55.2 |
| 290 | GSM36805 | ER+ | 100 | 0 | 45.6 | 8.3 |
| 292 | GSM36808 | ER- | 58 | 0 | -86.2 | -42.4 |
| 293 | GSM36809 | ER- | 56 | 0 | -111.0 | -121.5 |
| 416 | GSM37038 | ER+ | 60 | 1 | -23.4 | -1.6 |
| 540 | GSM36813 | ER+ | 49 | 1 | 28.6 | 14.9 |
| 546 | GSM36818 | ER+ | 62 | 1 | 56.3 | 61.5 |
| 550 | GSM36826 | ER+ | 58 | 1 | 29.6 | 29.5 |
| 600 | GSM36782 | ER+ | 66 | 0 | 103.2 | 82.1 |
| 601 | GSM36783 | ER+ | 52 | 0 | 95.9 | 74.3 |
| 602 | GSM36784 | ER+ | 57 | 1 | 45.9 | -32.3 |
| 605 | GSM36785 | ER+ | 57 | 0 | 78.5 | 32.8 |
| 606 | GSM36786 | ER+ | 66 | 0 | 59.5 | 68.2 |
| 609 | GSM36788 | ER- | 79 | 0 | -213.3 | -179.9 |
| 611 | GSM36799 | ER+ | 75 | 0 | 47.9 | 35.0 |
| 612 | GSM36802 | ER+ | 92 | 0 | 64.8 | 85.6 |
| 613 | GSM36803 | ER+ | 93 | 0 | 121.9 | 32.6 |
| 614 | GSM36804 | ER+ | 88 | 0 | 86.9 | 59.4 |
| 615 | GSM36806 | ER+ | 92 | 0 | 114.5 | 116.9 |
| 616 | GSM36807 | ER+ | 88 | 0 | 78.0 | 79.0 |
| 620 | GSM36811 | ER+ | 62 | 1 | 108.4 | 69.9 |
| 621 | GSM36812 | ER- | 80 | 0 | 97.1 | 77.9 |
| 625 | GSM36815 | ER+ | 72 | 1 | 129.7 | 108.2 |
| 627 | GSM36817 | ER+ | 113 | 0 | -33.5 | -37.5 |
| 629 | GSM36819 | ER+ | 131 | 0 | 67.2 | 46.8 |
| 630 | GSM36823 | ER+ | 119 | 0 | -53.9 | -26.1 |
| 631 | GSM36825 | ER+ | 99 | 0 | 115.0 | 76.2 |
| 633 | GSM36828 | ER- | 76 | 0 | -165.9 | -147.9 |
| 634 | GSM36830 | ER+ | 117 | 0 | 141.9 | 61.3 |
| 635 | GSM36832 | ER+ | 119 | 0 | 107.5 | 68.7 |
| 637 | GSM36833 | ER- | 54 | 0 | -135.6 | -121.8 |
| 640 | GSM36842 | ER+ | 81 | 0 | -25.0 | -18.7 |
| 641 | GSM36843 | ER+ | 77 | 0 | 49.8 | 36.7 |
| 642 | GSM36845 | ER+ | 54 | 0 | 139.6 | 126.7 |
| 646 | GSM36851 | ER+ | 100 | 0 | -34.0 | -16.9 |
| 647 | GSM36854 | ER- | 105 | 0 | 63.7 | 41.4 |
| 648 | GSM36857 | ER+ | 95 | 0 | 26.0 | 9.3 |
| 696 | GSM36960 | ER+ | 51 | 1 | -145.9 | -127.0 |
| 698 | GSM36963 | ER+ | 60 | 0 | 31.3 | 23.1 |
| 703 | GSM36814 | ER+ | 61 | 1 | 1.2 | -25.0 |
| 707 | GSM36816 | ER- | 98 | 0 | -18.7 | -37.6 |
| 710 | GSM36822 | ER- | 50 | 0 | -199.5 | -167.5 |
| 712 | GSM36824 | ER+ | 86 | 0 | -154.4 | -151.7 |
| 713 | GSM36827 | ER- | 156 | 0 | -171.3 | -193.7 |
| 714 | GSM36829 | ER+ | 157 | 0 | -78.1 | -53.9 |
| 716 | GSM36831 | ER+ | 87 | 0 | 184.9 | 153.1 |
| 726 | GSM36848 | ER+ | 105 | 0 | 183.3 | 165.3 |
| 727 | GSM36850 | ER+ | 121 | 0 | 74.4 | 74.1 |
| 728 | GSM36853 | ER+ | 105 | 0 | 107.2 | 64.6 |
| 729 | GSM36856 | ER+ | 86 | 0 | 74.9 | 71.6 |
| 737 | GSM36868 | ER+ | 123 | 0 | 178.5 | 171.7 |
| 738 | GSM36869 | ER+ | 126 | 0 | 155.8 | 134.4 |
| 741 | GSM36893 | ER+ | 124 | 0 | 48.7 | 35.5 |
| 747 | GSM36895 | ER+ | 96 | 0 | 13.5 | 19.6 |
| 749 | GSM36820 | ER+ | 141 | 0 | -54.1 | -67.6 |
| 751 | GSM36910 | ER+ | 125 | 0 | 114.8 | 114.3 |
| 754 | GSM36913 | ER+ | 109 | 0 | 96.2 | 46.9 |
| 760 | GSM36878 | ER+ | 98 | 0 | 24.1 | -13.8 |
| 762 | GSM36970 | ER+ | 116 | 0 | 149.5 | 96.1 |
| 763 | GSM36934 | ER+ | 171 | 0 | -66.6 | -85.8 |
| 765 | GSM36781 | ER+ | 147 | 0 | -136.4 | -116.7 |
| 767 | GSM36841 | ER+ | 134 | 0 | 70.9 | 25.2 |
| 768 | GSM36884 | ER+ | 129 | 0 | -59.6 | -76.0 |
| 769 | GSM36930 | ER+ | 84 | 0 | 39.6 | 4.2 |
| 772 | GSM37047 | ER+ | 108 | 0 | -162.7 | -150.0 |
| 774 | GSM36959 | ER- | 120 | 0 | -122.6 | -98.7 |
| 777 | GSM36961 | ER- | 153 | 0 | -106.8 | -93.3 |
| 778 | GSM36951 | ER+ | 104 | 0 | 85.8 | 87.6 |
| 779 | GSM37000 | ER+ | 137 | 0 | 112.0 | 72.3 |
| 780 | GSM36977 | ER- | 124 | 0 | -115.1 | -110.9 |
| 782 | GSM36978 | ER- | 148 | 0 | 15.8 | 30.9 |
| 783 | GSM36980 | ER+ | 122 | 0 | 82.6 | 70.2 |
| 785 | GSM36984 | ER+ | 138 | 0 | 69.9 | 5.9 |
| 787 | GSM36975 | ER+ | 110 | 0 | -24.8 | -31.7 |
| 789 | GSM36988 | ER+ | 96 | 0 | 102.5 | 94.1 |
| 791 | GSM36794 | ER+ | 87 | 0 | -15.0 | -24.0 |
| 793 | GSM37009 | ER+ | 122 | 0 | 51.8 | -1.5 |
| 794 | GSM37010 | ER+ | 101 | 0 | 64.6 | 40.7 |
| 795 | GSM37012 | ER+ | 132 | 0 | 154.1 | 124.7 |
| 796 | GSM37014 | ER+ | 143 | 0 | -29.8 | 12.8 |
| 797 | GSM37015 | ER+ | 122 | 0 | 142.2 | 78.5 |
| 798 | GSM36779 | ER+ | 132 | 0 | 85.2 | 54.4 |
| 804 | GSM36844 | ER+ | 121 | 0 | -102.0 | -93.9 |
| 805 | GSM36846 | ER- | 103 | 0 | -132.0 | -158.4 |
| 806 | GSM36847 | ER- | 85 | 0 | -240.9 | -204.6 |
| 808 | GSM36849 | ER+ | 110 | 0 | 117.2 | 43.6 |
| 809 | GSM36852 | ER+ | 107 | 0 | 70.7 | 11.4 |
| 810 | GSM36864 | ER+ | 84 | 0 | 39.7 | 41.8 |
| 813 | GSM36865 | ER- | 103 | 0 | -192.4 | -194.0 |
| 815 | GSM36866 | ER+ | 107 | 0 | 168.4 | 92.1 |
| 817 | GSM36867 | ER+ | 108 | 0 | 110.3 | 90.7 |
| 820 | GSM36890 | ER+ | 116 | 0 | -45.8 | -32.4 |
| 833 | GSM36912 | ER- | 97 | 0 | -258.2 | -229.5 |
| 836 | GSM36914 | ER+ | 84 | 0 | 58.5 | 57.9 |
| 839 | GSM36915 | ER- | 97 | 0 | -26.6 | -9.6 |
| 841 | GSM36932 | ER+ | 96 | 0 | 109.1 | 85.4 |
| 843 | GSM36933 | ER+ | 110 | 0 | -7.6 | -25.0 |
| 844 | GSM36935 | ER- | 114 | 0 | -174.9 | -140.2 |
| 845 | GSM36936 | ER+ | 109 | 0 | 113.2 | 63.1 |
| 846 | GSM36780 | ER- | 84 | 0 | -63.3 | -21.8 |
| 847 | GSM36810 | ER+ | 105 | 0 | 46.2 | 55.8 |
| 848 | GSM36840 | ER+ | 86 | 0 | 98.1 | 70.7 |
| 849 | GSM36883 | ER+ | 88 | 0 | 76.5 | 43.4 |
| 851 | GSM36945 | ER+ | 92 | 0 | 129.4 | 64.0 |
| 852 | GSM36995 | ER+ | 113 | 0 | -17.8 | -12.0 |
| 853 | GSM37034 | ER- | 88 | 0 | -97.1 | -64.4 |
| 855 | GSM36795 | ER- | 88 | 0 | -176.3 | -161.8 |
| 856 | GSM36821 | ER+ | 88 | 0 | -8.2 | -33.7 |
| 857 | GSM36863 | ER- | 98 | 0 | -14.7 | -33.2 |
| 861 | GSM37024 | ER+ | 95 | 0 | 127.0 | 74.7 |
| 862 | GSM37048 | ER- | 87 | 0 | -33.8 | -24.0 |
| 863 | GSM36979 | ER+ | 107 | 0 | 39.1 | 26.6 |
| 864 | GSM36981 | ER- | 87 | 0 | -171.6 | -164.4 |
| 865 | GSM36982 | ER+ | 92 | 0 | 92.1 | 75.6 |
| 866 | GSM36983 | ER+ | 75 | 1 | 124.4 | 107.7 |
| 867 | GSM36985 | ER+ | 55 | 1 | -29.4 | -0.2 |
| 868 | GSM36986 | ER+ | 77 | 1 | 87.9 | 87.7 |
| 870 | GSM37005 | ER+ | 56 | 1 | 111.5 | 87.5 |
| 871 | GSM37006 | ER+ | 71 | 1 | -13.4 | -15.7 |
| 873 | GSM37007 | ER+ | 59 | 1 | 44.9 | 29.1 |
| 874 | GSM37008 | ER+ | 70 | 1 | 75.4 | 56.4 |
| 876 | GSM37011 | ER+ | 60 | 1 | 58.2 | 10.3 |
| 877 | GSM37013 | ER+ | 66 | 1 | 58.3 | 3.4 |
| 879 | GSM37016 | ER- | 108 | 0 | -115.9 | -73.1 |
| 883 | GSM37032 | ER+ | 86 | 0 | 92.4 | 78.4 |
| 887 | GSM37033 | ER+ | 161 | 0 | 61.1 | 38.7 |
| 890 | GSM37043 | ER- | 161 | 0 | -163.2 | -152.0 |
| 891 | GSM37044 | ER+ | 112 | 0 | 61.1 | 47.3 |
| 894 | GSM37045 | ER- | 123 | 0 | -237.7 | -193.7 |
| 899 | GSM37046 | ER+ | 86 | 0 | 72.5 | 80.4 |
| 900 | GSM36887 | ER+ | 108 | 0 | 78.8 | 42.9 |
| 901 | GSM36889 | ER+ | 108 | 0 | 18.2 | 19.1 |
| 903 | GSM36892 | ER+ | 110 | 0 | -6.9 | 26.5 |
| 909 | GSM36894 | ER+ | 109 | 0 | 53.7 | 32.7 |
| 913 | GSM36911 | ER+ | 80 | 1 | 17.3 | 55.9 |

Table 2A Correlation of genes in Rosetta profile to 3-year prognosis

| Unigene | Name | Weight | P value  (one-sided RST) |
| --- | --- | --- | --- |
| Hs.55028 | CENPN | -4.35 | 3.55905E-05 |
| Hs.433512 | ACTR3 | -4.74 | 0.000350625 |
| Hs.188569 | ZDHHC13 | -3.8 | 0.000502425 |
| Hs.514527 | BIRC5 | -4.55 | 0.00094746 |
| Hs.350966 | PTTG1 | -3.85 | 0.001344573 |
| Hs.518464 | PSMD2 | -3.8 | 0.001509414 |
| Hs.445000 | PTGER3 | 5.51 | 0.004894351 |
| Hs.84113 | CDKN3 | -4.64 | 0.005388479 |
| Hs.495728 | PIR | -4.12 | 0.007182108 |
| Hs.592317 | TGFB3 | 4.02 | 0.012082112 |
| Hs.491148 | PCM1 | 3.74 | 0.014378721 |
| Hs.83383 | PRDX4 | -3.7 | 0.017510901 |
| Hs.567267 | FANCA | -3.8 | 0.020063449 |
| Hs.547696 | NUP155 | -4 | 0.022305647 |
| Hs.155204 | ZNF174 | 4.12 | 0.023700841 |
| Hs.433951 | GPX4 | 3.8 | 0.030270635 |
| Hs.567410 | PSMD14 | -4.2 | 0.035060423 |
| Hs.190518 | C21orf45 | -3.92 | 0.037429667 |
| Hs.173162 | COX4NB | -4 | 0.038391572 |
| Hs.576154 | LRP8 | -3.7 | 0.042380907 |
| Hs.153752 | CDC25B | -3.92 | 0.042466721 |
| Hs.436187 | TRIP13 | -3.74 | 0.044409293 |
| Hs.2006 | GSTM3 | 4.55 | 0.049282876 |
| Hs.532803 | HN1 | -3.7 | 0.050355459 |
| Hs.153357 | PLOD3 | -3.66 | 0.080824549 |
| Hs.65758 | ITPR3 | -3.74 | 0.0848953 |
| Hs.517830 | BTD | 3.92 | 0.090697491 |
| Hs.484738 | MYLIP | 3.92 | 0.099153471 |
| Hs.469649 | BUB1 | -4.2 | 0.100744195 |
| Hs.113876 | WHSC1 | -3.66 | 0.106321278 |
| Hs.429 | ATP5G3 | -4.52 | 0.106997655 |
| Hs.79353 | TFDP1 | -4.11 | 0.11309047 |
| Hs.547509 | SMARCE1 | 4.62 | 0.123444421 |
| Hs.492618 | EXT1 | -4 | 0.146572916 |
| Hs.268787 | ARIH1 | -4.7 | 0.153776456 |
| Hs.30743 | PRAME | -4.68 | 0.199852739 |
| Hs.390729 | ERBB4 | 3.8 | 0.227202562 |
| Hs.473648 | GART | -4.39 | 0.22952613 |
| Hs.508716 | COL4A2 | -3.74 | 0.242347758 |
| Hs.523468 | SCUBE2 | 6.57 | 0.242482242 |
| Hs.148767 | RQCD1 | -5.4 | 0.413936495 |
| Hs.81934 | ACADSB | 3.8 | 0.429861066 |
| Hs.77448 | ALDH4A1 | 3.85 | 0.469424166 |
| Hs.521012 | FLJ21062 | 4.29 | 0.51739725 |
| Hs.20013 | SYF2 | 4.66 | 0.524175322 |
| Hs.380164 | KRTAP4-7 | -4.72 | 0.553417256 |
| Hs.591190 | PEX12 | 4.07 | 0.692576004 |
| Hs.496068 | PCTK1 | -5.12 | 0.796132851 |
| Hs.439200 | KIAA0090 | 4.7 | 0.892460695 |
| Hs.518475 | EIF4A2 | -4 | 0.901649176 |
| Hs.128425 | C19orf29 | 4 | 0.948464931 |
| Hs.352962 | LOC285086 | -5.09 | NA |
| Hs.520974 | YWHAG | -5.05 | NA |
| Hs.9589 | UBQLN1 | -5.15 | NA |
| Hs.482233 | DEPDC1B | -4.25 | NA |
| Hs.35096 |  | 4.14 | NA |
| Hs.512963 | ALG11 | 4 | NA |
| Hs.408062 | KLC4 | 3.92 | NA |
| Hs.173034 |  | 3.85 | NA |
| Hs.308045 | NCAPH | -3.7 | NA |

Table 2B Correlation of genes in combined profile to 3-year prognosis

| Unigene | Name | Weight | P value  (one-sided RST) |
| --- | --- | --- | --- |
| Hs.435326 | ACTL6A | -3.91 | 2.20202E-06 |
| Hs.226390 | RRM2 | -3.99 | 1.64109E-05 |
| Hs.69771 | CFB | 4.08 | 2.53163E-05 |
| Hs.58974 | CCNA2 | -4.17 | 3.30674E-05 |
| Hs.567352 | TXNRD1 | -4.21 | 6.97883E-05 |
| Hs.78619 | GGH | -4.29 | 0.000172517 |
| Hs.188569 | ZDHHC13 | -4.43 | 0.000426287 |
| Hs.433512 | ACTR3 | -4.13 | 0.000866179 |
| Hs.350966 | PTTG1 | -3.96 | 0.001277871 |
| Hs.514527 | BIRC5 | -4.6 | 0.002031991 |
| Hs.409065 | FEN1 | -4.02 | 0.002257572 |
| Hs.632299 | NUP205 | -4.26 | 0.003004086 |
| Hs.495728 | PIR | -3.96 | 0.004203405 |
| Hs.491148 | PCM1 | 4.79 | 0.005653696 |
| Hs.83383 | PRDX4 | -4.36 | 0.006012191 |
| Hs.445000 | PTGER3 | 4.79 | 0.006790132 |
| Hs.436912 | KIFC1 | -3.95 | 0.009836367 |
| Hs.78771 | PGK1 | -4.14 | 0.01203964 |
| Hs.3416 | ADFP | -4.45 | 0.013735866 |
| Hs.374378 | CKS1B | -4.07 | 0.014879082 |
| Hs.513797 | SLC7A5 | -4.14 | 0.01881182 |
| Hs.173162 | COX4NB | -4.42 | 0.022417801 |
| Hs.292579 | PTDSS1 | -4.36 | 0.027200736 |
| Hs.150749 | BCL2 | 4.59 | 0.028457233 |
| Hs.190518 | C21orf45 | -4.69 | 0.02910337 |
| Hs.153752 | CDC25B | -4.47 | 0.02949687 |
| Hs.532803 | HN1 | -4.34 | 0.033975516 |
| Hs.436187 | TRIP13 | -4.48 | 0.035793105 |
| Hs.2006 | GSTM3 | 4.62 | 0.040859494 |
| Hs.498661 | USP6NL | -4.16 | 0.045169081 |
| Hs.524399 | TROAP | -4.16 | 0.055540167 |
| Hs.153357 | PLOD3 | -3.98 | 0.063440739 |
| Hs.492618 | EXT1 | -4.17 | 0.06848128 |
| Hs.12272 | BECN1 | 4.92 | 0.072731759 |
| Hs.111554 | ARL4C | -4.41 | 0.087631853 |
| Hs.576154 | LRP8 | -4.76 | 0.091447785 |
| Hs.267659 | VAV3 | 5.01 | 0.098761166 |
| Hs.483444 | CXCL14 | 4.37 | 0.110519835 |
| Hs.469649 | BUB1 | -4.56 | 0.11618426 |
| Hs.79353 | TFDP1 | -4.32 | 0.125264731 |
| Hs.524134 | GATA3 | 4.93 | 0.125670474 |
| Hs.65758 | ITPR3 | -4.24 | 0.171426483 |
| Hs.523468 | SCUBE2 | 5.18 | 0.221338328 |
| Hs.371013 | JMJD2B | 4.94 | 0.254769073 |
| Hs.197320 | TLE1 | -4.3 | 0.33519392 |
| Hs.81934 | ACADSB | 4.68 | 0.33519392 |
| Hs.473648 | GART | -3.88 | 0.354015223 |
| Hs.208124 | ESR1 | 5.23 | 0.383665881 |
| Hs.584836 | ITGBL1 | 4.38 | 0.397246466 |
| Hs.82906 | MPL | -4.42 | 0.423998574 |
| Hs.496068 | PCTK1 | -4.94 | 0.701877504 |
| Hs.12109 | CIAO1 | -4.07 | NA |
| Hs.35096 |  | 5.14 | NA |
| Hs.520974 | YWHAG | -4.94 | NA |
| Hs.308045 | NCAPH | -4.5 | NA |
| Hs.9589 | UBQLN1 | -3.94 | NA |
| Hs.482233 | DEPDC1B | -3.9 | NA |
| Hs.5719 | NCAPD2 | -4.1 | NA |
| Hs.530735 | MS4A7 | 4.74 | NA |
| Hs.486401 | C6orf173 | -3.99 | NA |
